# The DNA-binding protein HU is a molecular glue that attaches bacteria to extracellular DNA in biofilms

**DOI:** 10.1016/j.jbc.2021.100532

**Published:** 2021-03-11

**Authors:** Bhishem Thakur, Kanika Arora, Archit Gupta, Purnananda Guptasarma

**Affiliations:** Centre for Protein Science, Design and Engineering (CPSDE), Department of Biological Sciences, Indian Institute of Science Education and Research (IISER) Mohali, Knowledge City, Punjab, India

**Keywords:** Nucleoid associated protein, extracellular DNA, bacterial biofilm, histone-like DNA-binding protein HU, lipopolysaccharide, bacterial clumping, protein-based charge neutralization, bacterial cell-DNA binding glue, explosive bacterial lysis, 5CB, 4-cyano-4′-pentylbiphenyl, c-LPS, cell-displayed LPS, CMC, critical micellar concentration, DAS, difference absorption spectroscopy, DIC, differential interference contrast, DMOAP, N,N-dimethyl-N-octadecyl-3-aminopropyl trimethoxysilyl chloride, e-DNA, extracellular DNA, f-HU, free HU, f-LPS, free LPS, FSC, forward scatter, HU, highly abundant nucleoid-associated histone-like protein, i-LPS, immobilized LPS, LC, liquid crystal, LPS, lipopolysaccharide, MST, microscale thermophoresis, NAP, nucleoid-associated protein, Ni-NTA, Nickel-Nitrilotriacetic acid, PI, propidium iodide, RFP, red fluorescent protein, SSC, side scatter, 4WJ, four-way junction

## Abstract

In biofilms, bacteria that possess a negatively charged surface are embedded within a matrix of polymers consisting mainly of negatively charged extracellular DNA (e-DNA). In all likelihood, a multivalent positively charged substance, for example, a basic protein, exists within biofilms to neutralize charge–charge repulsions and act as a ‘glue’ attaching negatively charged bacteria to negatively charged e-DNA; however, no protein capable of doing so has yet been identified. We decided to investigate whether a highly abundant nucleoid-associated histone-like protein (HU) happens to be the glue in question. In recent years, HU has been shown to possess qualities that could be considered desirable in the proposed glue, for example, (a) availability in association with e-DNA; (b) multivalent DNA binding; (c) non–sequence-specific DNA-binding; (d) enhancement of biofilm formation upon exogenous addition, and (e) disruption of biofilms, upon removal by HU–cognate antibodies. Geometric considerations suggest that basic residues in HU's canonical and noncanonical DNA-binding sites can interact with sugar-linked terminal phosphates in lipopolysaccharide (LPS) molecules in bacterial outer membranes. Here, using genetic, spectroscopic, biophysical–chemical, microscopy-based, and cytometry-based experiments, we demonstrate that HU's DNA-binding sites also bind to LPS, that this facilitates DNA–DNA, DNA–LPS, and LPS–LPS interactions, and that this facilitates bacterial clumping and attachment of bacteria to DNA. Exogenous addition of HU to bacteria in (nonshaken) cultures is shown to cause cells to become engulfed in a matrix of DNA, potentially arising from the lysis of bacteria with vulnerable cell walls (as they strain to grow, divide, and move away from each other, in opposition to the accreting influence of HUs).

Bacteria in biofilms are embedded within a matrix of extracellular polymeric substances, consisting mainly of extracellular DNA (e-DNA) released through cell lysis (1), or cellular secretions (2). A conceptual problem with the embedment of bacteria in a matrix of DNA is that the surfaces of both bacteria and DNA naturally tend to be highly negatively charged because of the presence of phosphate groups in both DNA's phosphodiester backbone and the lipid A head groups of lipopolysaccharide (LPS) molecules on the outer membranes of gram-negative bacteria. In gram-positive bacteria, in place of LPSs, negatively charged lipoteichoic acid molecules are present upon bacterial surfaces. Therefore, regardless of the type of bacterium involved, DNA and bacteria are expected to repel one another, within biofilms (3). The fact that they coexist thus indicates the presence of some sort of a charge-neutralizing molecular glue that binds to both bacteria and DNA.

In theory, any molecular glue capable of binding to both DNA and cells could be a protein that is decorated with positive charges on its surface (4). Also, in theory, such a protein could be any protein displaying some level of nonspecific binding to DNA and cells. However, an ideal molecular glue would be one that is capable of binding to both cells and DNA with reasonable specificity and comparable affinity.

We begin this article by arguing that the ideal molecular glue in a biofilm would need to have the following sets of characteristics: (i) it would need to be a protein capable of binding to DNA both abundantly and ubiquitously, to ensure that e-DNA displays a uniformly charged, positively charged surface. Furthermore, (ii) the protein would need to be multivalent, multimeric, and present in the extracellular medium (either by itself or in association with e-DNA), to ensure its binding to both cells and e-DNA through multiple sites on its surface. Satisfaction of these two sets of criteria could allow a protein to bind to both cells and e-DNA in various modes, allowing the protein to act as a noncovalent cross-linker of cells and e-DNA.

To the best of our understanding, from among all the DNA-binding proteins present in bacteria, the ideal candidate satisfying every one of the above requirements is the highly abundant nucleoid-associated histone-like protein (HU), for the following reasons: HU is a highly abundant, DNA-binding, histone-like, nucleoid-associated protein (NAP) of the DNABII class. HU has already been reported to be present in bacterial biofilms (5). HU has also been discovered to be limiting for biofilm formation, that is, anti-HU antibodies have been demonstrated to disrupt biofilms (6). HU is known to be ubiquitously present upon DNA, on account of its binding to DNA in a non–sequence-specific manner (7). HU is known to be one of the most abundant proteins present in bacteria, reaching levels of up to 50,000 dimers per cell during exponential growth (8). HU is known to be released into the extracellular medium through multiple mechanisms, including lysis of dead cells (1), explosive cell lysis (9), and secretion (2) by mechanisms including type IV secretion (10, 11, 12). HU is known to be multivalent in its ability to bind to DNA (13, 14, 15), both because HU dimers associate into higher oligomers and because each HU dimer within any HU oligomer itself hosts four motifs for DNA binding [including two (canonical) lysine/arginine-rich beta hairpins, and two (noncanonical) double-lysine clusters]. Thus, the protein, HU, fulfills every conceivable criterion for being the molecular glue that attaches cells to DNA and to other cells.

Because HU is a known DNA-binding protein, it remains to be established (a) whether HU is capable of binding to LPS molecules, and to cells, and (b) whether HU is capable of causing agglomeration of LPS molecules, and of cells. In *Escherichia coli*, HU exists as two isoforms, HU-A and HU-B, which are both ∼90 amino acids long (16) and share approximately ∼69% sequence identity. HU-A is synthesized mainly in the late lag and early log phases of growth and exists predominantly as a dimer or tetramer, whereas HU-B is synthesized mainly in the mid-log phase and exists as a dimer, tetramer, or octamer (13, 16, 17). In the late-log phase and stationary phase, it is believed that heterodimers of HU-A and HU-B exist in combination with DNA (18).

In a previous article, we have described engineered forms of HU-A and HU-B, in which each protein has been genetically fused with a fluorescent protein at its N-terminus (19). In that article, we had created chimeric forms of HU in which a monomeric red fluorescent protein (tag-RFP) was fused with HU-A (to make RFP–HU-A). Similarly, a monomeric yellow fluorescent protein (Venus) was fused with HU-B (to make Venus–HU-B). In the work described here, which explores the binding of HU to LPS and DNA, we have used both 6xHis-tagged and affinity-purified WT forms of HU-A and HU-B, without any fluorescent protein in fusion, and also with a fluorescence protein in fusion (*i.e.*, RFP–HU-A and Venus–HU-B), as well as certain truncated, protein-engineered forms of HU, in addition to HU in which specific phenylalanine residues have been replaced by the fluorescent residue, tryptophan (Trp).

In this article, using a combination of spectroscopic, microscopic, cytometric, and other investigations, we show that HU-A and HU-B, as well as variants of these isoforms of HU, bind to free LPS (f-LPS) and LPS on the outer membranes of bacterial cells (cell-displayed LPS [c-LPS]). We show that the binding of HU to f-LPS or c-LPS involves either, or both, of HU's canonical, or noncanonical, DNA-binding sites. We show that addition of micellar f-LPS to free HU (f-HU) generates large molecular assemblies of LPS. We also show that addition of f-HU to cells generates large cellular assemblies (or bacterial clumps).

The charged head group present in the lipid A component of LPS contains two hexose-linked terminal phosphate moieties. Each of these bears at least one, and potentially two, positive charges (based on the pH of the environment and the degree of ionization). We propose that these phosphate moieties bind to the lysine/arginine residues positioned on (each of) f-HU's two types of DNA-binding sites, through charge–charge interactions and molecular recognition involving specific distance and geometry restraints. We demonstrate that LPS and DNA are able to compete with each other for binding to the HU. Thus, we propose that HU may have evolved to bind to both DNA and LPS. Our findings do not discount the possibility that other DNA-binding proteins might also prove to act as molecular glues in biofilms, in future studies.

## Results

### Structural bioinformatics–based exploration of possible binding geometries of LPS to HU's canonical and noncanonical DNA-binding sites

**Figure 1:**
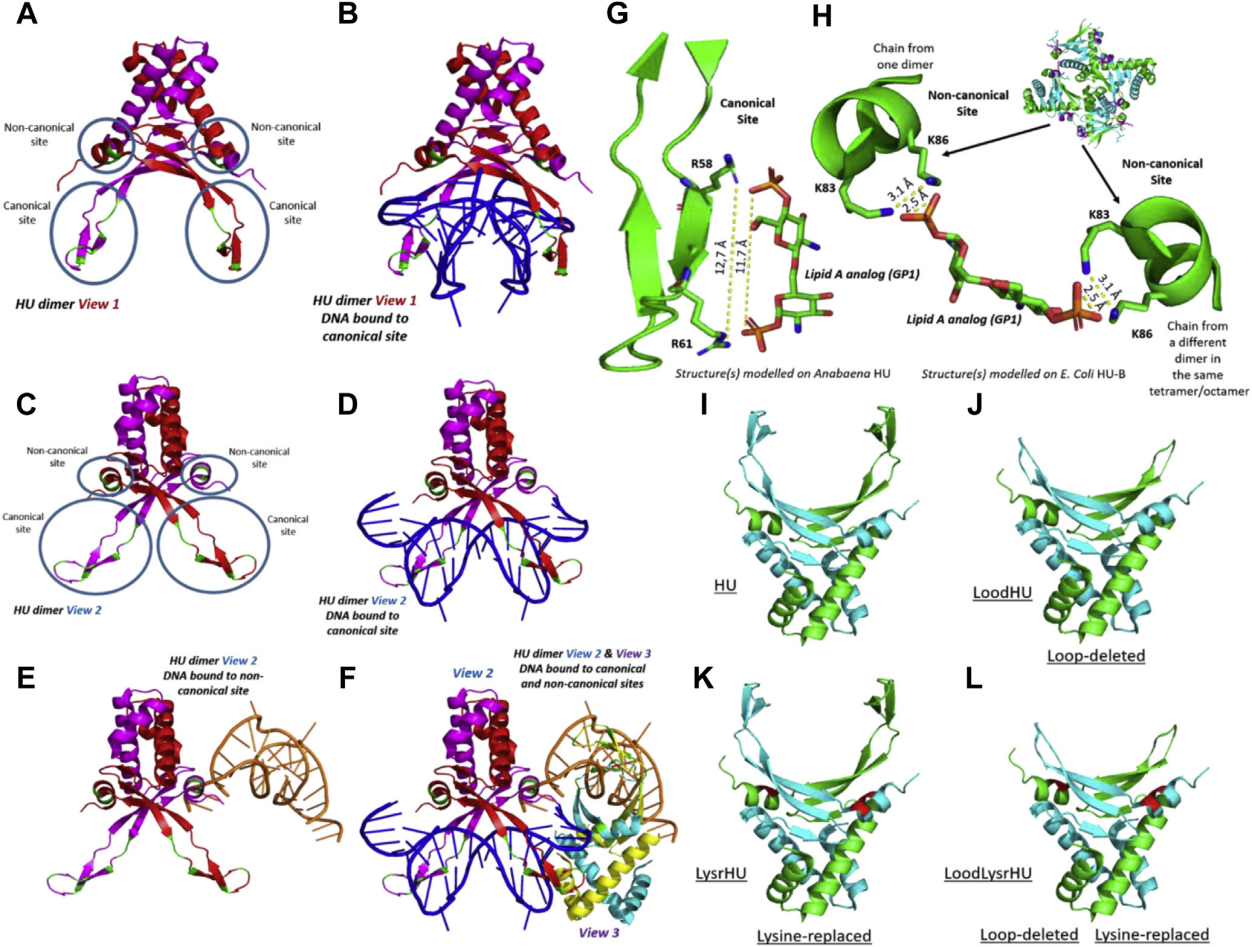
**HU's canonical and noncanonical interactions with DNA and potential modes of interaction with LPS: variants created to probe interactions with DNA and LPS**. *A*, an HU dimer (view 1; *Anabaena* HU; derived from PDB ID: 1P51) showing locations of the canonical and noncanonical DNA-binding sites. *B*, the same HU shown in (*A*) (view 1) with a 20-mer double-stranded DNA molecule bound at its canonical DNA-binding site. *C*, the same HU shown in panel A, but rotated by 45 degrees to obtain a different view (view 2). *D*, the same (DNA-bound) HU shown in (*B*), but rotated by 45 degrees (view 2), clearly showing the beta hairpin loops from each of the two monomers grasping DNA around the minor groove. *E*, the same HU shown in (*C*), but with the canonical site unoccupied and with a 20-mer double-stranded DNA molecule bound at the noncanonical DNA binding site. *F*, the same HU shown in (*C–E*), but with both canonical and noncanonical DNA-binding sites occupied by 20-mer double-stranded DNA molecules; in this arrangement, two HU dimers from a single asymmetric unit of the crystal are shown (one from view 2 and the other from a different view, view 3); each dimeric HU's canonically bound DNA is also bound to the noncanonical site of the other dimeric HU. *G*, a proposal based on the structure of *Anabaena* HU (PDB ID: 1P51) regarding the use of two conserved arginine residues (R58 and R61) on a beta hairpin loop from the canonical DNA-binding site for binding to the sugar-phosphate head group of the lipid A component of lipopolysaccharide (LPS); here, a *stick* representation of the epsilon amino groups of arginine is used to establish distance compatibility with the two terminal phosphate groups in the head group of lipid A. *H*, a proposal based on the structure of *E. coli* HU (PDB ID: 2O97) regarding the use of two conserved lysine residues (K83 and K86) on a helix on the side of each monomer of HU for binding to a doubly charged terminal phosphate in the sugar-phosphate head group of the lipid A component of LPS, with the other terminal phosphate bound to a different helix from a different dimer in an HU higher-order multimer. I, WT HU with both canonical and noncanonical DNA-binding sites present. *J*, a schematic of loop-deleted HU (LoodHU) in which the canonical DNA-binding sites are deleted and replaced with an 11-amino acid-long glycine/serine-rich linker. *K*, a schematic of lysine-replaced HU (LysrHU) in which lysines K83 and K86 at the noncanonical DNA-binding sites (shown in *red*) are replaced by alanine. *L*, a schematic of loop-deleted and lysine-replaced HU (LoodLysrHU), in which both canonical and noncanonical DNA-binding sites are missing. The PDB ID 1P51 was used to generate the schematics in panels I-L. HU, highly abundant nucleoid-associated histone-like protein.

Figure 1*A* shows, in green color, the polypeptide backbone locations of the canonical DNA binding site(s) in HU that are present on each of the two chains of an HU dimer. The two sites, determined through X-ray crystallography (14), are shown upon the structure of the *Anabaena* HU dimer. Figure 1*A* also shows the noncanonical DNA-binding site(s) that were identified later, using small-angle X-ray scattering studies of the structurally analogous *E. coli* HU (15). The reason that Figure 1*A* shows *Anabaena* HU instead of *E. coli* HU, for which the X-ray crystallographic structure is also known (20), is that electron density data are currently only available for the canonical DNA-binding loops of *Anabaena* HU in the presence of DNA, and not for *E. coli* HU (presumably owing to mobility of these loops in the absence of DNA).

Figure 1*B* shows the *Anabaena* HU structure in complex with a double-stranded 20-mer fragment of DNA (in blue) bound at the canonical DNA-binding site. Figure 1, *A* and *B* represent HU from the same viewing angle (view 1) and are derived from a crystal in which two dimers of *Anabaena* HU display crystallographic association; basically, each 20-mer (HU-bound) DNA fragment is also bound to a different HU dimer within the asymmetric unit, through the terminal regions of the DNA fragment.

In the next few panels of Figure 1, we use these additional noncovalent interactions to schematically illustrate how HU can simultaneously also bind to DNA through HU's noncanonical DNA-binding sites. First, in Figure 1*C*, we show the HU dimer of Figure 1*A* from a different angle (view 2) rotated by 45 degrees in respect of view 1, along a vertical axis. Correspondingly, Figure 1*D* shows the DNA-bound view of the rotated HU dimer of Figure 1*C*. Figure 1, *E* and *F* display interactions of HU with DNA at the noncanonical site. Using view 2, in Figure 1*E*, a different (second) copy of the double-stranded 20-mer DNA present in the same asymmetric unit (bound to the canonical site of a different dimer) can be seen to be interacting with the noncanonical site of the dimer shown in Figure 1, *A*–*D* (in yellow).

Finally, in Figure 1*F*, a complete view of both dimers in the asymmetric unit is shown, with each dimer associating with one copy of the 20-mer double-stranded DNA at its canonical site, and with a different copy of the 20-mer double-stranded DNA at its noncanonical site. In this figure, the second HU dimer is shown from a third view (view 3), which is different from view 1 and view 2. Figure 1, *A*–*F* could therefore be taken to be schematic diagrams, that is, diagrams that use real crystal structures, and known and determined interactions of *Anabaena* HU with DNA to illustrate how the analogous *E. coli* HU could also potentially simultaneously interact with DNA at both its canonical and noncanonical sites.

Based on the above structural–biochemical perspectives, we decided to theoretically examine whether HU's DNA-binding sites could also potentially bind to negatively charged LPS molecules, either through HU's binding to LPS in the form of f-LPS that has been shed into the extracellular medium or to c-LPS that is still in association with bacterial outer membranes. We examined distances between negatively charged phosphate moieties in LPS and positively charged lysine/arginine residues present at the canonical and noncanonical DNA-binding sites. The head group of the lipid A component of LPS contains two hexose-linked phosphate moieties.

There are two important differences between these moieties and the analogous sugar–phosphate moieties in DNA. First, the sugars in DNA/RNA are pentose sugars (deoxyribose or ribose), whereas those in the lipid A head group of LPS are hexose sugars (glucosamine). Second, the phosphate groups in the DNA backbone contain only one negative charge per phosphate because of the participation of the phosphate in the formation of phosphodiester bonds; in contrast, in the head group of lipid A, both phosphates are terminal phosphates and, therefore, could potentially possess two negatively charged oxygen atoms each, based upon the pH of the environment. The anticipated pKa values of the two OH groups in a terminal phosphate moiety are 2.2 and 7.2. At a neutral pH, of course, only one of these (the one with a pKa of 2.2) would be expected to carry a charge. However, at higher values of pH, some molecules could potentially host two negative charges, that is, the lipid A head group could exist as either H_2_PO_4_^−^, with a single negative charge, or as HPO_4_^2−^, with two negative charges, in environments in which the pH is unregulated and higher than 7.2. For example, it is quite well known that the pH of LB medium is routinely around 9.0, and also that further alkalization may occur in the absence of glucose, because consumption of glucose leads to the acidification of the medium. Therefore, it may thus be conjectured that at a pH of 9.0, phosphates in the lipid A group of LPS would host either two charges or at least a partial additional charge on the OH group with a pKa of 7.2. Even in PBS, with a pH of 7.4, the possibility of the existence of two charges on terminal phosphates remains. Therefore, the distinction between the terminal phosphates of the lipid A group of LPS, and the phosphates of the DNA backbone, could very well be significant.

Notably, the phosphates on the lipid A group of LPS are already known to bind to poly-L-lysine through electrostatic interactions (21). Based on similar interactions, Figure 1, *G* and *H* schematically show how these phosphates could also potentially bind to lysine/arginine groups present at the DNA-binding sites of HU, using the structures of the polypeptide backbones for segments of the canonical (Fig. 1*G*) and noncanonical (Fig. 1*H*) DNA-binding sites of *Anabaena* HU. In these figures, the side chains of the relevant lysine and arginine residues have been shown in green (stick model). The backbones of the HU segmental structures have been shown juxtaposed against the known crystal structure of the head group of lipid A, sourced from the crystal structure of an antibody bound to the same head group (22).

Figure 1*G* shows how the two phosphate groups in the lipid A component of LPS (one each at the two ends of the head-group) can potentially bind to either one or even two different positively charged, ε-amino groups (separated by 12.7 Å) located on arginine residues, R58 and R61, in HU's canonical DNA-binding site. The two arginine residues, R58 and R61, are conserved between *Anabaena* and *E. coli* HU, and also in the HU from most other bacteria (23). Figure 1*H* shows how two negative charges present on the terminal phosphate group present at any end of the head group of lipid A could potentially bind to one or even two ε-amino groups (separated by 3.1 Å) located on two lysine residues, K83 and K86, on the same face of a helix in HU's noncanonical DNA-binding site. Together, Figure 1, *G* and *H*, therefore, suggest that a theoretical case exists for LPS to bind to HU, not merely through nonspecific electrostatic interactions but potentially through specific interactions between phosphate groups and lysine/arginine side chains. The distance restraints considered also indicate some degree of molecular recognition between LPS HU.

### HU binds to the f-LPS

Below, we describe six different kinds of biophysical–chemical experiments, which demonstrate that the purified recombinant HU and/or recombinant RFP–HU-A bind to *E. coli–*derived f-LPS.

#### Microscale thermophoresis: Binding of f-LPS affects RFP–HU-A diffusion in temperature-jump experiments in capillaries

**Figure 2:**
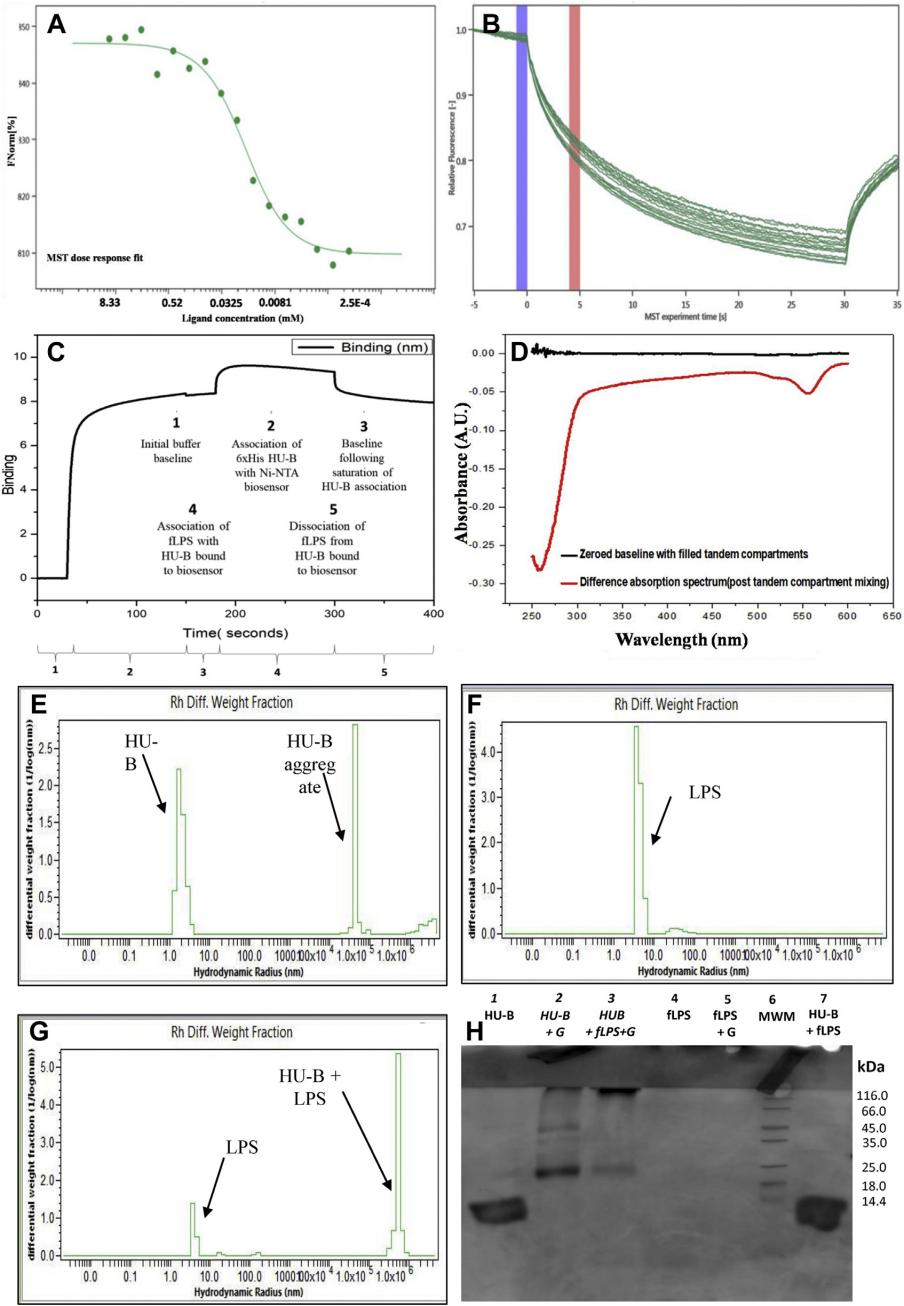
**Binding of HU to f-LPS.***A*, microscale thermophoresis (MST) dose–response curve for the binding of f-LPS by HU-A, based on varying concentrations of f-LPS and a fixed concentration of fluorescent tag-RFP–HU-A and plotting rates of reduction in fluorescence from tag-RFP–HU-A due to (temperature jump dependent) diffusion of molecules away from a microscopic volume of the solution being monitored inside a capillary, with different capillaries used for different f-LPS concentrations. *B*, raw microscale thermophoresis data traces for 16 capillaries containing different concentrations of f-LPS; *blue* and *red bars* indicate the beginning and ending of the time period used to calculate normalized rates of reduction in tag-RFP–HU-A fluorescence for capillaries over a 30-seconds-long laser-induced temperature jump; each curve plots the fall in fluorescence in a separate capillary, and the subsequent rise, over 5 s, after heating is switched off. *C*, biolayer interferometry (BLI) sensorgram showing the washing baseline (segment 1), binding of HU-B to the Ni-NTA-derivatized sensor (segment 2), washing baseline (segment 3), binding of f-LPS to HU-B (segment 4), and washing-based dissociation (segment 5). *D*, difference absorption spectroscopy (DAS) data monitoring binding of HU-B to f-LPS after zeroing of baseline; premixing spectra (*black*) and postmixing spectra (*red*) are used to detect hyperchromic/hypochromic effects in HU-B phenylalanine (∼265 nm) and RFP chromophore (550 nm) absorption bands due to binding. *E–G*, dynamic light scattering (DLS) monitoring changes in the size of 0.02-μm–filtered HU-B, f-LPS, and HU-B + f-LPS to detect noncovalent crosslinking of f-LPS by HU-B. *H*, denaturing SDS-PAGE investigating covalent (glutaraldehyde-mediated) crosslinking of HU-B by f-LPS. f-HU, free HU; f-LPS, free LPS; HU, highly abundant nucleoid-associated histone-like protein; LPS, lipopolysaccharide; RFP, red fluorescent protein.

Figure 2, *A* and *B* show a titration of RFP–HU-A against varying concentrations of micellar and nonmicellar f-LPS, ranging from 2.5 mg/ml to 0.038 μg/ml, spanning the critical micellar concentration (CMC) of LPS which is ∼10 μg/ml. This was performed to examine whether the rate of diffusion of RFP–HU-A is altered because of binding of f-LPS to HU-A and also whether there is any difference in the behavior noticed between pre-CMC and post-CMC concentrations of f-LPS. The diffusion of RFP–HU-A either involves motion of molecules away from or back toward a region of ‘temperature jump–induced’ molecular depletion within a set of capillaries containing identical HU and differing LPS concentrations. The MST experiment monitors the dose dependence of the rate of reduction of fluorescence (through diffusion-aided depletion), as well as the rate of increase of fluorescence (through diffusion-aided replenishment, after restoration of the temperature) of the RFP fluorescence signal. The experiment demonstrates that the diffusion of RFP–HU-A, both away from a region of temperature jump–induced depletion of molecules and back toward the same region, tends to be progressively slower for higher concentrations of f-LPS. This indicates that RFP–HU binds to micellar f-LPS. The data also show no discontinuity at the 10 μg/ml boundary concentration, between pre-CMC and post-CMC concentrations of f-LPS, suggesting that the lipid A moiety in f-LPS remains equally accessible for binding of HU in nonmicellar and micellar forms of f-LPS (where the micellar form is a simulacrum for cell-surface LPS).

The estimated binding constant from these studies would appear to be ∼34 μM, if one assumes binding of one LPS ligand molecule per HU chain monomer, with an average molecular weight of 15,000 Da for f-LPS. Of course, both of these assumptions do not hold because (i) all f-LPS molecules do not have the exact same molecular weight, (ii) the LPS concentrations used are in the post-CMC range, and (iii) the HU population consists of multiple quaternary structural forms, and not HU monomers. Therefore, the value of ∼34 μM must only be taken to be indicative of the low-affinity nature of the binding of HU to LPS and not as an accurate measure.

#### Biolayer interferometry: f-LPS binds to HU-B to generate sensor grams

Figure 2*C* shows sensor grams for binding of 6xHis affinity-tagged HU-B onto a Nickel-nitrilotriacetic acid (Ni-NTA) derivatized probe and for binding of f-LPS to this Ni-NTA–bound 6xHis-tagged HU-B [similar to sensor grams obtained in surface plasmon resonance experiments but detected here using a different and analogous technique called biolayer interferometry (BLI)]. An association of the histidine-tagged HU-B with the Ni-NTA–derivatized probe tip is observed and so is a subsequent association of micellar f-LPS with the tip-bound HU-B. The micellar f-LPS is observed to cause the LPS-HU association curve to descend even during the association phase, presumably because of distortions caused by progressively greater binding of micellar f-LPS which could cause the bound molecular masses to shift away from the probe tip's surface, or even cause some leaching. However, this effect seen during association is quite small in comparison with the dissociation response that results from dissociation of f-LPS and HU, following depletion of f-LPS from the solution. This indicates that HU-B binds to f-LPS.

#### Difference absorption spectroscopy: binding of f-LPS alters the UV-visible absorption spectra of RFP–HU-A

Figure 2*D* shows ‘instrument-zeroed’ absorption profiles of premixing (black) and postmixing (red) states for an experiment in which equal volumes of RFP–HU-A and f-LPS were mixed. In this experiment, RFP–HU-A and micellar f-LPS are initially present in separate compartments of a split-quartz (tandem-compartment) cuvette with a separating wall rising to two-thirds of the cuvette's height, allowing light to pass through both compartments in the control experiment, and also mixing of the contents of the two compartments to be effected through inversion of the cuvette (after closing of the lid), for the subsequent experiment examining interaction between the constituents of the two compartments. Mixing of equal volumes of potentially interacting species, followed by refilling of both compartments through ‘uprighting’ of the cuvette, results in the halving of concentrations of the species in each compartment, and the doubling of the path length of light passing through solutions of RFP–HU-A and f-LPS (because both species fall back into both compartments, after the mixing of the contents of the two compartments through uprighting of the cuvette). A difference in absorbance of light passing through both compartments is anticipated if, and only if, there are interactions between the species originally present in the two compartments, after mixing. This interaction manifests as a deviation(s) from the zero baseline because of alterations of the electronic states of chromophores adjacent to interacting surfaces due to the intermolecular interactions. If there are no interactions, no difference in absorbance is expected on account of the Beer–Lambert law, as it applies to a difference absorption spectroscopy (DAS) experiment because there is a halving of concentrations and a doubling of path lengths (24, 25). In the experiment shown in Figure 2*D*, mixing is clearly shown to produce deviations manifesting as bands corresponding to reduction in the aromatic absorption of HU around ∼260 nm because of changes in absorptivity of HU's phenylalanine residues (note: HU contains no Trp or tyrosine residues) and also around ∼550 nm because of reduction in absorption of the tag-RFP chromophore. These two negative bands in the DAS (red) spectrum at ∼260 and ∼550 nm show that RFP–HU-A does indeed bind to micellar f-LPS.

#### Dynamic light scattering: HU-B crosslinks nonmicellar (filtered) f-LPS into large noncovalent assemblies

**Figure 3:**
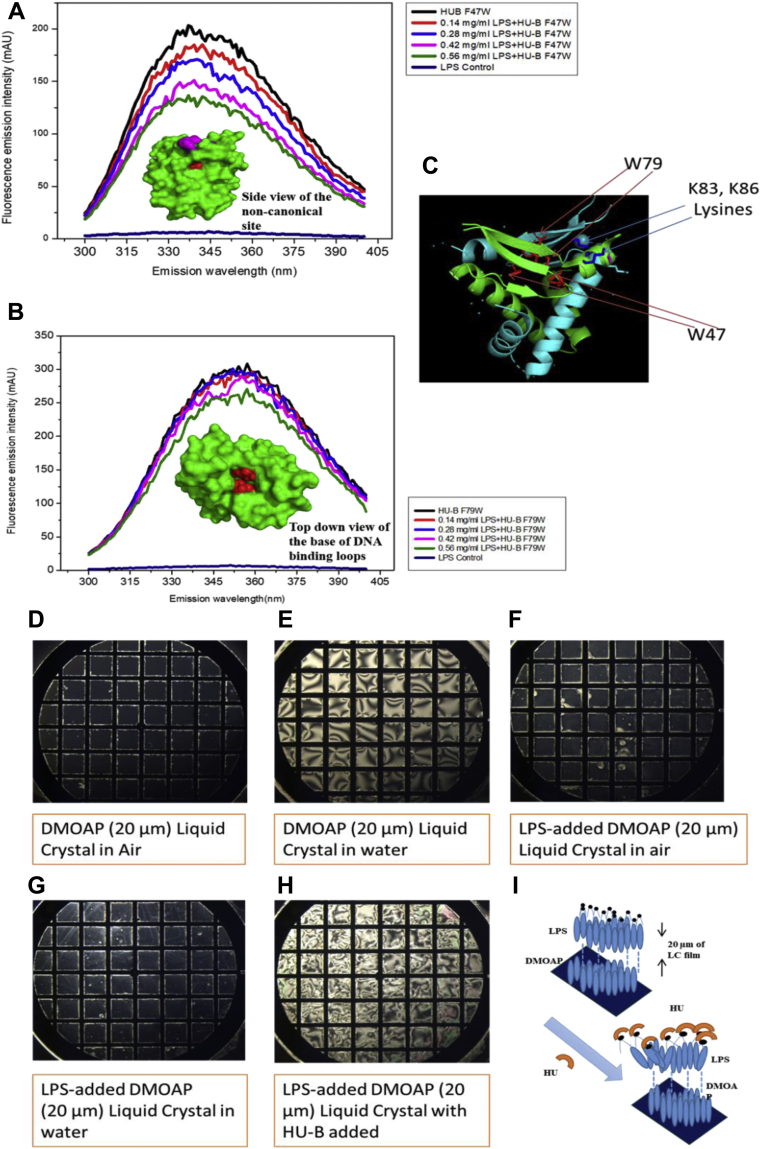
**Binding of HU to f-LPS and i-LPS.***A*, quenching of tryptophan fluorescence in mutant (F47W) HU-B by f-LPS binding; in the inset, residue W47 from one monomer is shown in *red* and seen to be located proximal to the noncanonical DNA-binding site residues K83 and K86, shown in *magenta*. *B*, quenching of tryptophan fluorescence in mutant (F79W) HU-B by f-LPS binding; in the inset, residue F79, from each of two monomers (facing each other) is shown in *red*, lying at the base of the canonical DNA-binding site. *C*, a ribbon diagram representation of the structure of the HU-A–HU-B heterodimer (PDB ID: 2O97) with superimposed *stick* representations of residues F47 and F79 (sites of mutations F47W and F79W) as well as residues K83 and K86.Optical polarization micrographs of birefringence from liquid crystals without i-LPS *D*, in air; *E*, under water; with i-LPS *F*, in air; *G*, under water; and *H*, upon addition of HU-B. *I*, a schematic *cartoon* showing the mechanism of changes in birefringence of the liquid crystal upon interaction of HU-B with i-LPS. f-LPS, free LPS; HU, highly abundant nucleoid-associated histone-like protein; i-LPS, immobilized LPS.

Figure 3 shows light-scattering profiles. These establish that HU-B (with an average size of ∼3–4 nm; Fig. 2*E*), upon addition to a population of f-LPS (with an average size of ∼8–9 nm; Fig. 2*F*), containing a minority population of micellar LPS (with an average size of ∼75–80 nm; Fig. 2*F*), generates large molecular assemblies (with sizes in the range of 5 × 10^5^ nm; Fig. 2*G*) that are an order of magnitude larger in size than aggregates of HU (with an average size of 4 × 10^4^ nm; Fig. 2*E*). Concomitantly, there is reduction of populations corresponding to both HU and f-LPS. Such molecular assemblies are anticipated to form upon binding of negatively charged f-LPS to HU-B because the protein exists as a variety of multimers (dimers, tetramers, and octamers) in which each dimer could have two (or more) sites of interaction with negatively charged LPS. Effectively, HU appears to noncovalently ‘crosslink’ f-LPS into large assemblies. It must be noted that in these specific (DLS) experiments, unlike in all the other experiments described in this section, the f-LPS solution was filtered through a 0.02-nm filtration device before light scattering. Therefore, we do not see the significant presence of f-LPS micelles (75–80 nm diameter) in Figure 2*F*, as already noted, that is, we happen to be operating predominantly in the pre-CMC range of LPS concentrations in which the filtered f-LPS has a diameter of about 8 to 9 nm, consisting of monomeric LPS or very small LPS aggregates. This shows that HU-B also binds to f-LPS in the nonmicellar form, and not just to the micellar form of LPS, as already shown in previous sections.

#### Glutaraldehyde addition: Glutaraldehyde covalently crosslinks HU-B and micellar f-LPS into very large assemblies

Figure 2*H* shows that upon incubation of HU-B with micellar f-LPS and glutaraldehyde (lane 3), the original HU monomer population in an SDS-PAGE (lane 1) disappears and is replaced by an assembly comprising crosslinked f-LPS–HU-B, which is so large that it is unable to enter into the resolving gel in the SDS-PAGE, after traversing the stacking gel (lane 3). Only a residual faint population of dimeric HU-B is observed. In contrast, glutaraldehyde itself has no comparable effect (lane 2) on HU alone, that is, only dimeric and tetrameric populations of the HU are stabilized through glutaraldehyde cross-linking in the absence of f-LPS, with some crosslinking leading to stabilization of trimers, and with the monomeric band being no longer seen (indicating that all dimers have at least one interchain crosslink). Furthermore, no band of intensity comparable with that seen in lane 3 is observed in lane 2, at the stacking-resolving gel interface. This indicates that glutaraldehyde-mediated crosslinking of HU-B does not generate large crosslinked assemblies comparable with those generated through cross-linking of HU-B to f-LPS. The control in lane 5 shows that no band is seen with the addition of glutaraldehyde to f-LPS; obviously, this is because the stain (Coomassie) does not bind to f-LPS. The control in lane 7 shows that without glutaraldehyde present to effect a covalent cross-linking, the noncovalently crosslinked assemblies of HU-B and f-LPS are dissociated by the effects upon HU-B on addition of SDS and boiling. Thus, lane 7 is identical to lane 1 because HU-B is seen to be predominantly monomeric because of the presence of SDS (and f-LPS is not stained by Coomassie). These experiments visually establish that HU-B and f-LPS form large assemblies that become covalently crosslinked by glutaraldehyde into objects that no longer penetrate the stacking-resolving gel interface of an SDS-PAGE.

#### Fluorescence quenching: f-LPS binding quenches fluorescence in HU-B Trp-containing mutants

Figure 3, *A* and *B* show the effects upon Trp fluorescence emissions in two Trp-containing mutants of HU-B, F47W HU-B and F79W HU-B, respectively, which fold correctly and retain DNA-binding ability (Fig. S1) upon addition of micellar f-LPS. The F79W position lies just under the beta hairpin constituting the canonical DNA-binding site in HU-B. The F47W position, in contrast, lies close to the lysine cluster constituting the noncanonical DNA-binding site. The spectra establish that there is less quenching of Trp fluorescence achieved by addition of micellar f-LPS to F79W HU-B than by addition of micellar f-LPS to F47W HU-B. The reasons for this differential response are evident from the differential degrees to which the Trp residues lie near the DNA-binding sites (see insets in Fig. 3, *A* and *B*) in the two mutants. This quenching suggests that the binding of micellar f-LPS to the HU's noncanonical DNA-binding site (proximal to F47W) elicits more of a response than binding to the canonical site (proximal to F79W), but that there is a response seen with both mutants. The data thus shows that HU-B binds to micellar f-LPS.

#### Changes in birefringence: binding of HU-B to immobilized LPS causes disordering of i-LPS–surfaced liquid crystals

Figure 3 shows effects of binding of HU to liquid crystal (LC)–immobilized LPS (i-LPS) upon birefringence of ordered LCs of N,N-dimethyl-N-octadecyl-3-aminopropyl trimethoxysilyl chloride (DMOAP) coated with 4-cyano-4′-pentylbiphenyl (5CB). In experiments conducted according to protocols established in previous studies (26), we show that the 5CB-coated DMOAP LCs exist in an ordered state when in the bulk phase and exposed to air, displaying a dark field under a polarizing microscope, as seen in Figure 3*D*. When the 5CB-coated DMOAP LCs are placed under water, the water causes a disordering transition in the hydrophobic LC, and a consequent conversion from dark field to bright field, as seen in Figure 3*E*. However, when micellar f-LPS is immobilized into becoming i-LPS (*i.e.*, immobilized f-LPS) upon the LCs of 5CB-coated DMOAP, the LCs once again undergo an ordering transition from bright field to dark field under a polarizing microscope, as shown in Figure 3*F*, in an air-exposed state. This arrangement shows no further alteration upon being placed under water, as seen in Figure 3*G*, because the LPS is charged at the end facing the water and its hydrophobic lipid tail (which interacts with the 5CB) is no longer affected by the water. Thereafter, binding of any reagent to the i-LPS can theoretically elicit a disordering transition that causes the LCs to go back from dark field to bright field even in water. This possibility is exploited to use the system as a sensor in an assay for the binding of any protein to i-LPS, as already known (26). In the present set of experiments, such a disordering transition from dark field to bright field is observed upon addition of 0.5 mg/ml HU-B to LC- i-LPS, as seen in Figure 3*H*, demonstrating that HU-B binds to i-LPS. Figure 3*I* shows a schematic for the overall disordering transition caused by protein binding to i-LPS.

### HU binds to c-LPS and induces bacterial clumping (which is inhibited by DNA, f-LPS, and salt)

#### c-LPS–HU–c-LPS interactions: Flow cytometry–based evidence of clumping of bacteria through binding of HU-B/HU-A to c-LPS

**Figure 4:**
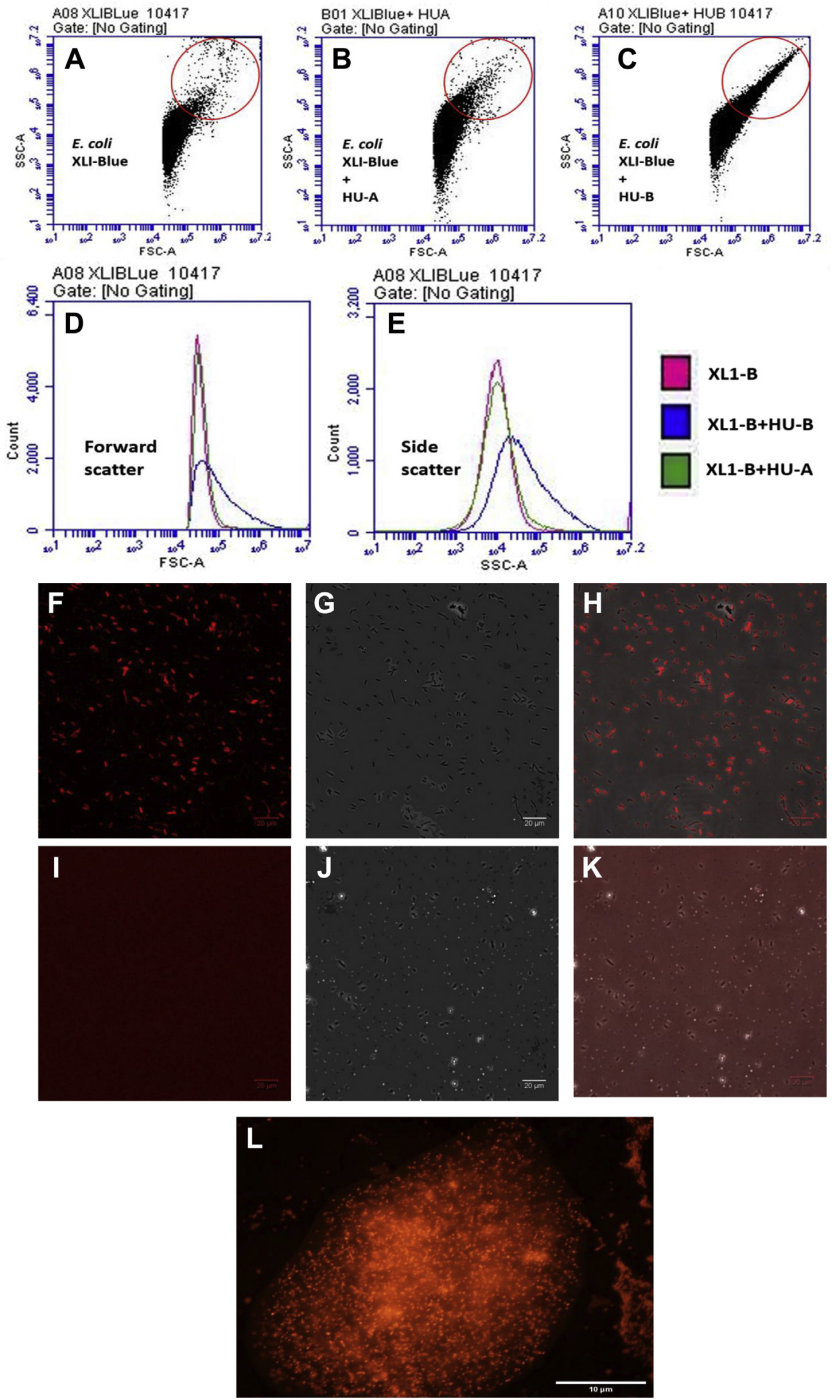
**HU–c-LPS and c-LPS–HU–c-LPS interactions.** Scatter plots derived from flow cytometry of *E. coli* cells, with monitoring of forward scatter *versus* side scatter using *A*, control XL1-Blue cells, *B*, XLI-Blue cells treated with HU-A, and *C*, XL1-Blue cells treated with HU-B. The *ovals* (*red outlines*) represent scatter plot areas with heightened forward and side scatter indicative of bacterial cell clumping. The streak represents clumped/aggregated cells. Distinctions between clumping caused by HU-A and HU-B are observed in (*D*), the combined overlay of cell count *versus* forward scatter, and *E*, the combined overlay of cell count *versus* side scatter. *F*, fluorescence, *G*, phase contrast, and *H*, merged images of XL1-Blue cells treated with exogenously added tag-RFP–HU-A. *I*, fluorescence, *J*, phase contrast, and *K*, merged images of XL1-Blue cells treated with only Tag-RFP. *L*, fluorescence image showing embedment of bacterial cells in a network of e-DNA created by growing cells without shaking in the presence of HU-B. c-LPS, cell-displayed LPS; e-DNA, extracellular DNA; HU, highly abundant nucleoid-associated histone-like protein; RFP, red fluorescent protein.

Figure 4, *A*–*E* show flow cytometry data that indicate that in the presence of HU-B, as well as HU-A, *E. coli* cells display increased forward scatter (FSC) as well as increased side scatter (SSC) profiles that are diagnostic of an increase in size through cell clumping. The clumping observed here is entirely similar to that observed when poly-D-lysine is added to *E. coli* cells to deliberately cause their clumping (Fig. S2). The clumping manifests as a streak on the top-right section of the FSC-SSC scatter plot. Figure 4*A* shows the FSC-SSC plots for control *E. coli* XL-1 Blue cells. When HU-A (Fig. 4*B*) or HU-B (Fig. 4*C*) are added to the *E. coli* cells, there is a tendency for streaks (arising from increased FSC and SSC) to be seen at the top right corner, due to bacterial clumping. When all other conditions are identical, HU-B appears to cause more clumping than HU-A in these FSC *versus* SSC plots. The distinction between the effect of HU-A and HU-B is also clearly evident in the cell counts plotted against FSC (Fig. 4*D*) and SSC (Fig. 4*E*), that is, it can be seen that (i) populations seen with addition of HU-A (in green) are only somewhat shifted with respect to the control, unlike populations seen with addition of HU-B (in blue), and (ii) a much more distinctive effect is seen for HU-B that for HU-A in the SSC plots, than in the FSC plots.

#### HU–c-LPS interactions: Fluorescence microscopic and flow cytometry–based evidence of binding of RFP–HU-A to bacteria

Figure 4, *F*–*K* show fluorescence micrographs establishing the binding of RFP–HU-A to the surfaces of planktonic bacteria in an isotonic buffer of pH 7.4. Control HU-A is nonfluorescent, as it lacks the presence of genetically fused tag-RFP. Therefore, no images are shown for this control. The addition of RFP–HU-A to cells of *E. coli* strain XL-1 Blue elicits localized fluorescence (Fig. 4*F*), which happens to be colocalized with the bacteria seen in the differential interference contrast (DIC) image (Fig. 4*G*) and in the merged image (Fig. 4*H*). In contrast, addition of RFP alone to *E. coli* cells elicits no such detectable localized RFP fluorescence under the same conditions (Fig. 4*I*) at locations corresponding to bacterial cells in the DIC image (Fig. 4*J*). Therefore, no overlap of fluorescence and DIC images is seen in the merged image (Fig. 4*K*). This establishes that it is the HU component of the RFP–HU-A fusion construct, rather than the RFP component, which causes RFP–HU-A to colocalize with bacterial cells. It is thus shown that HU binds to the surfaces of bacterial cells and not to something inside the cells (because cells were not permeabilized for this experiment). The indication is that HU binds to the outer membrane LPS (c-LPS), with the RFP domain present in the RFP–HU-A fusion highlighting the titration of the HU upon bacterial cell surfaces.

It may be noted that the above experiments involved cells that do not overexpress any recombinant HU. In separate experiments that involved the use of a wide-field fluorescence microscope with capabilities of gathering in-plane images with live bacteria, with the stacking of these images into a three-dimensional representation allowing examination of fluorescence within bacteria and outside bacteria, we also observed that cells that overexpress recombinant Venus–HU-B (which sometimes remain syncytial, and form long filaments, while sometimes breaking into smaller filaments and cells) stick to each other and display a halo of Venus fluorescence outside the cell surface, in addition to the Venus fluorescence associated with the bacterial genomic nucleoid in the cell cytoplasm. (Videos in Fig. S3).

In flow cytometry experiments, when RFP–HU-A was added to cells, rather than to HU-A or HU-B alone, the RFP labeled the cell surface on all cells, and clumps, that display RFP fluorescence (as can be seen in Fig. S4). However, RFP–HU-A itself causes less clumping like HU-A, and the streak is smaller than with HU-B, as previously noted during the use of HU-A to clump cells.

The above observations reconfirm the ability of the HU to bind to bacterial cell surfaces through binding to c-LPS. In addition, they show that when HU forms large multimeric assemblies, it can cause clumping of bacteria by using different LPS-binding surfaces on such multimers to bind to different bacteria. It is conceivable that when RFP is present in fusion with HU at HU's N-terminus, this might sterically prevent the formation of tetramers and octamers by dimeric HU polypeptides, although the presence of the RFP as a domain in the fusion clearly does not interfere with the binding of RFP–HU-A to cells, as was already observed in Figure 4, *F*–*K*.

#### c-LPS–HU–c-LPS and c-LPS–HU–DNA–HU–c-LPS interactions: Fluorescence microscopic imaging of bacteria embedded in a matrix of e-DNA

We have already alluded to the generation of e-DNA through cell lysis (1), in particular, through explosive cell lysis during which rod-shaped bacteria lose their shapes and become spheres, which then break up to release cytoplasmic contents and DNA, which is then rapidly disseminated in a population of proximally growing bacterial cells (9). It is possible that the rapid dissemination of such released e-DNA by bacteria occurs through the binding of the e-DNA to bacterial cell surfaces, with the assistance of multivalent and abundant NAPs such as HU. This suggests that addition of HU to a population of growing cells could increase the binding of cells to each other and to e-DNA, promoting greater amounts of explosive cell lysis and generation of even higher amounts of e-DNA.

We found that when bacteria are grown without shaking in the presence, and absence, of exogenously added HU-B, large bacterial clumps are generated in the nonshaking petri plates to which HU-B is added. Figure 4*L* shows that when propidium iodide (PI), a DNA-binding dye, is added to such plates after permeabilization of bacteria, and clumps are imaged for PI fluorescence, the bacteria (fluorescing due to genomic DNA-bound PI) are observed to be embedded in a matrix of e-DNA (fluorescing due to e-DNA–bound PI). In Figure 4*L*, therefore, fluorescent bacteria are seen to be embedded in a matrix of DNA which is also fluorescent. This shows that HU (and other proteins performing a similar function) could play a key role in explosive cell lysis, through creation of physical (noncovalent) links between cells and other cells, and between cells and e-DNA, promoting a greater tendency for lysis of cells through physical stress. Such physical stress, exerted upon growing cells, could use weaknesses generated in the cell wall at the sites of cell wall growth, in addition to weaknesses in membranes, and cause explosive lysis. Our data thus suggests that the availability of any HU in the extracellular medium can promote the generation of e-DNA, and the triggering of a feedback mechanism through which more e-DNA leads to even more e-DNA, facilitating embedment, growth of colonies, and lysis of cells.

#### f-LPS–HU, DNA–HU, and c-LPS–HU interactions: Flow cytometry–based evidence for inhibition of c-LPS–HU interactions by DNA and f-LPS

**Figure 5:**
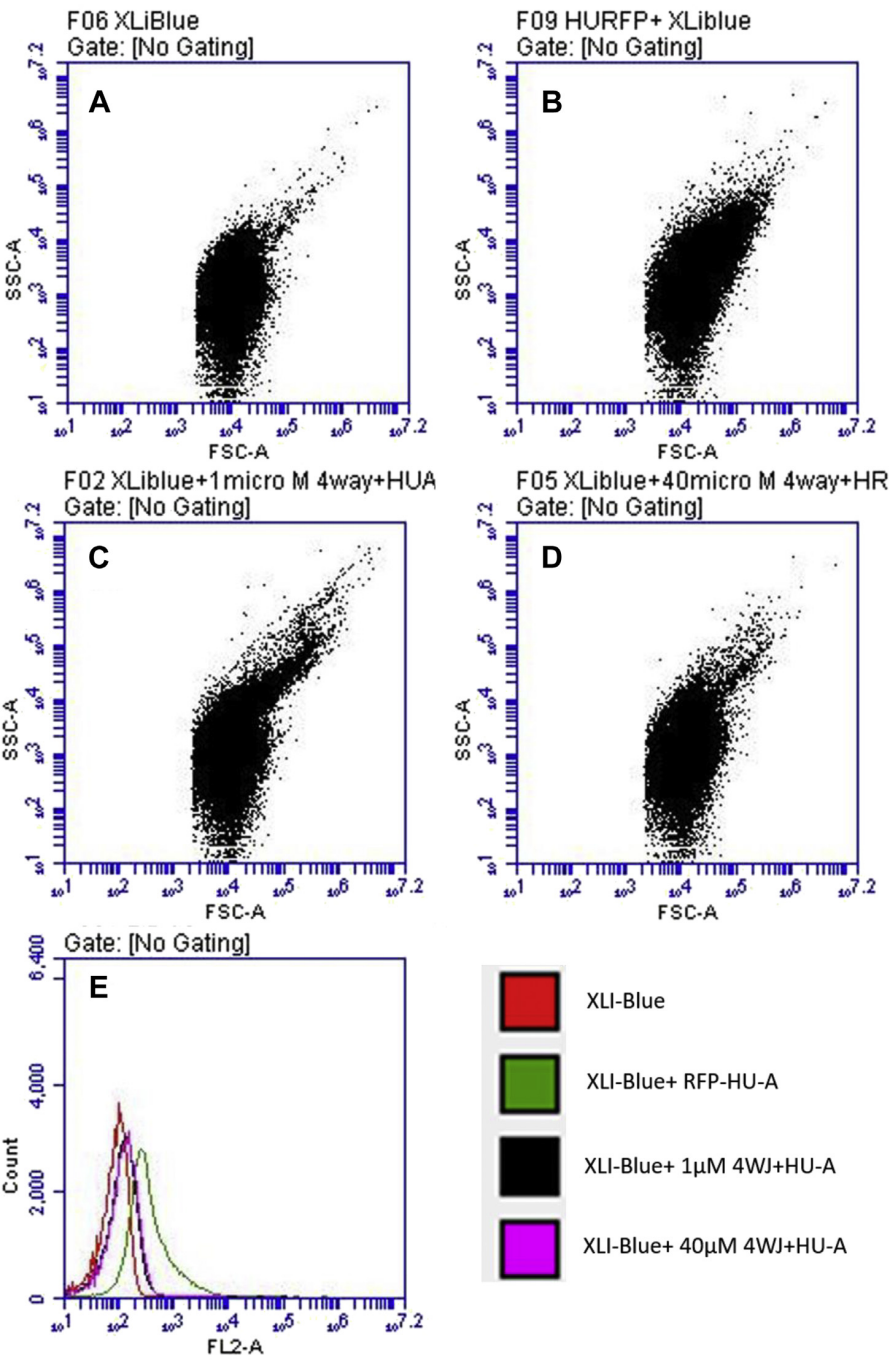
**Inhibition of c-LPS–HU–c-LPS interactions (*E. coli* binding and clumping) through preincubation of Tag-RFP–HU-A with DNA (4WJ).** DNA dose-dependent reduction in intensity of streaks in scatter plots derived from flow cytometry of *E. coli* cells, with monitoring of forward scatter *versus* side scatter using *A*, control XL1-Blue cells, *B*, XL1-Blue cells treated with Tag-RFP–HU-A, *C*, XL1-Blue cells treated with Tag-RFP–HU-A pretreated with 1-μM 4WJ DNA, *D*, XL1-Blue cells treated with Tag-RFP–HU-A pretreated with 40-μM 4WJ DNA. *E*, combined overlay of fluorescence from Tag-RFP–HU-A bound to cells with (and without) pretreatment with 4WJ DNA; saturation of binding sites on HU-A which are capable of binding to either DNA or c-LPS, by DNA, is observed. c-LPS, cell-displayed LPS; HU, highly abundant nucleoid-associated histone-like protein; LPS, lipopolysaccharide; RFP, red fluorescent protein; 4WJ, four-way junction.

Figure 5 shows FSC *versus* SSC scatter plots, similar to those shown in Figure 4, *A*–*E*, showing a dose-dependent reduction of HU-mediated *E. coli* cell clumping, which is observed upon preincubation of RFP–HU-A with four-way junction (4WJ) DNA. This suggests that an excess of DNA available for prebinding to HU saturates its binding sites and reduces the scope for use of such sites for binding of the HU to c-LPS, upon cell surfaces.

In an analogous set of experiments involving preincubation of RFP–HU-A with f-LPS, instead of with 4WJ DNA, Figure S5 shows that there is a similar reduction in *E. coli* cell clumping, as the concentration of f-LPS is increased from 0 mg/ml (Fig. S5; top left panel) to 0.5 mg/ml (Fig. S5; top right panel) to 1 mg/ml (Fig. S5; bottom left panel). This suggests that an excess of f-LPS made available for prebinding to HU saturates HU's LPS-binding sites and thus reduces the scope for the use of such sites for binding of the HU to c-LPS. In other words, f-HU molecules that are not prebound to something else (*e.g.*, DNA or f-LPS) are better at causing *E. coli* cell clumping through multivalent binding to c-LPS than f-HU molecules which are prebound to either DNA or f-LPS. It must be borne in mind, of course, that these experiments are suggestive. The actual dissociation constants of f-LPS, c-LPS, and 4WJ DNA, for HU-A, and the relative concentrations of c-LPS (dependent upon the number of cells) remain undetermined. These would, of course, be likely to influence quantitative aspects of the actual data seen.

#### c-LPS-HU interactions: Flow cytometry–based evidence of inhibition of c-LPS-HU interactions by NaCl

Figure S6 shows control cells (left top panel), cells in the presence of HU-B (right top panel), and cells in the presence of HU-B with two different concentrations of NaCl (bottom left and right panels). These indicate that NaCl is able to progressively screen out HU–LPS interactions, sufficient to abolish the streak indicative of clumping.

### Variants of HU lacking either canonical or noncanonical sites (but not both) bind to DNA and LPS

To examine the interactions of DNA and LPS with HU, we created several protein-engineered HU variants. These variants are depicted in Figure 1, *I–L*. The variants are called LoodHU, LysrHU, and LoodLysrHU and were created for HU-A and HU-B. The canonical DNA-binding site on an HU dimer consists of two loops, one derived from each HU monomer, as shown in Figure 1, *A* and *G*, and the noncanonical DNA-binding sites consist of a double-lysine cluster on each monomer, consisting of lysine residues K83 and K86, as shown in Figure 1, *A* and *H*. We created four different forms of HU-A and HU-B: (A) HU itself (shown in Fig. 1*I*) containing both canonical and noncanonical DNA-binding sites, (B) loop-deleted HU, or LoodHU (as shown in Fig. 1*J*), in which the 22-residue-long loop (extending from residue 52 to residue 74 in both HU-A and HU-B) was deleted and replaced by an 11-residue-long, glycine/serine-rich linker peptide (N-SGGGGSGGGGS-C), to ablate the canonical DNA-binding loop/site, (C) lysine-replaced HU, or LysrHU (as shown in Fig. 1*K*), in which lysine residues, K83 and K86, were both replaced by the residue, alanine, to ablate/remove the noncanonical DNA-binding site, and (D) loop-deleted and lysine-replaced HU, or LoodLysrHU (as shown in Fig. 1*L*), in which both canonical and noncanonical DNA-binding sites were ablated through genetic manipulation. In addition, we also made variants in which mutations K83A and K86A were made individually.

#### Characterization of LoodHU-B, LysrHU-B, and LoodLysrHU-B through comparison with WT HU-B

The WT HU and its three variants were created and compared with each other in respect of their ability to fold into dimeric HU. Figure S7 shows CD spectra establishing that HU, and all three of the variants created to ablate DNA-binding sites, that is, LoodHU, LysrHU, and LoodLysrHU, have comparable structural contents with mean residue ellipticity values in the range of -9000 to -9500 deg cm^2^ dmol^−1^ at 208 nm, and negative mean residue ellipticity bands at 208 nm and 222 nm, arising from the helical content of these forms of HU. Figure S8 shows gel filtration data which establish that the HU, LoodHU, LysrHU, and LoodLysrHU all have gel filtration elution profiles in which the HU elutes at ∼12 ml, corresponding to a molecular weight of approximately 25 kDa (resulting from dimerization of two ∼10.6-kDa HU chains, each made up of a ∼1.4-kDa affinity tag [N-MRGSHHHHHHGS] and a ∼9.2-kDa HU polypeptide, with some disorder in the beta hairpin DNA-binding loop adding to the protein's hydrodynamic volume).

#### 4WJ DNA binds to HU-B, LoodHU-B, and LysrHU-B but not to LoodLysrHU-B

**Figure 6:**
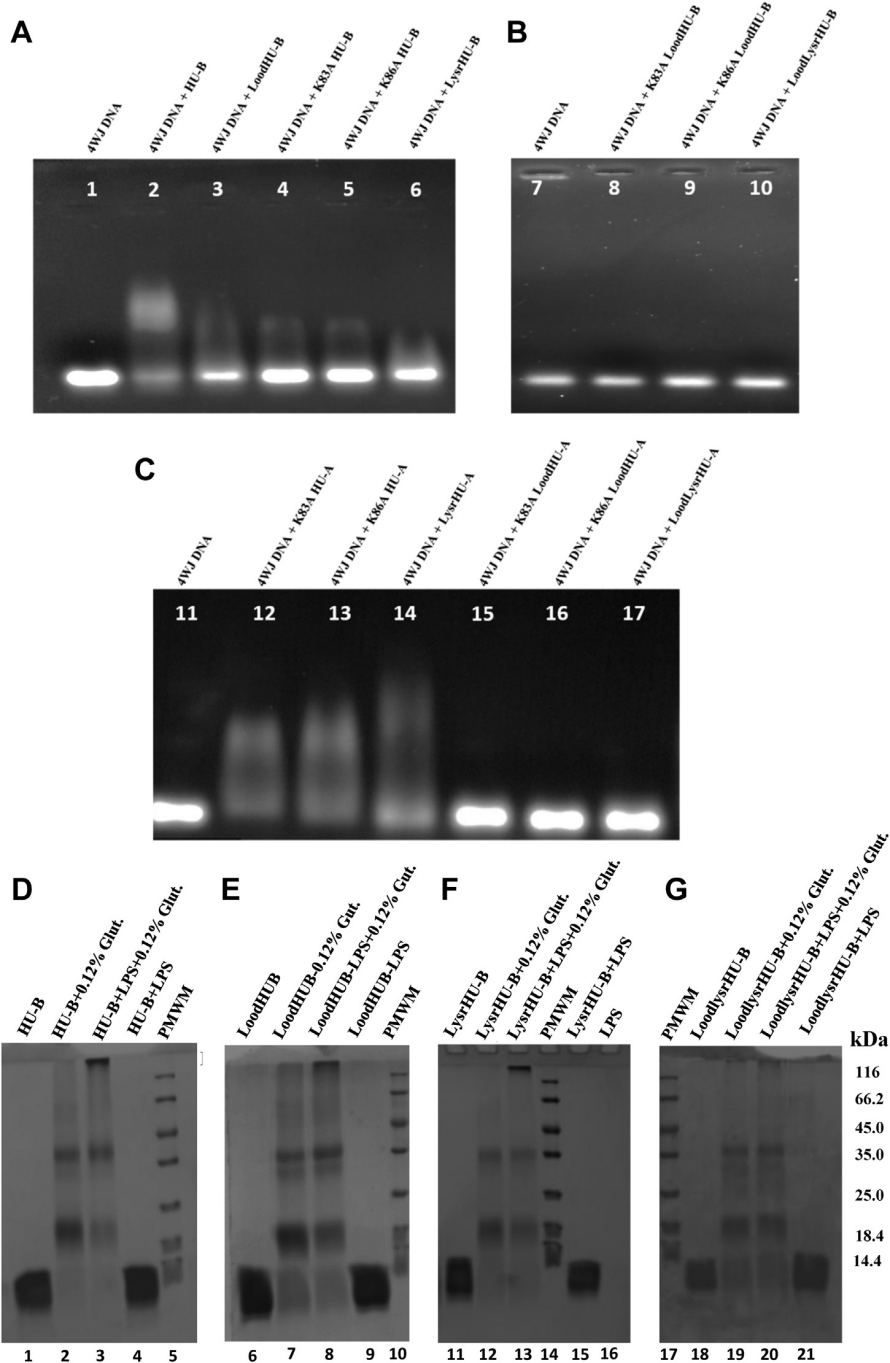
**Binding of 4WJ DNA and f-LPS by HU-B, HU-A, and their Lood, Lysr, and LoodLysr variants**. Electrophoretic mobility shift assay (EMSA) performed on agarose gels with EtBr-stained four-way junction (4WJ) DNA bound to various HU variants as shown in (*A*), for individual HU-B variants with the ablated canonical site, or partially/totally ablated noncanonical site. *B*, for HU-B variants with the ablated canonical site and partially/totally ablated noncanonical site, or *C*, for individual HU-A variants with the ablated canonical site or partially/totally ablated noncanonical site, or HU-A variants with both the ablated canonical site and partially/totally ablated noncanonical site. SDS-PAGE gel assays for glutaraldehyde-based covalent crosslinking of HU-B into HU-B multimers (dimers, tetramers, and hexamers) and HU-B–f-LPS high molecular weight (HMW) forms unable to cross the stacking-resolving gel interface, as shown in (*D*), for HU-B. *E*, for loop-deleted HU-B with the ablated canonical site; *F*, for lysine cluster-deleted HU-B with the ablated noncanonical site; *G*, for HU-B with the combined ablated canonical and ablated noncanonical sites. f-LPS, free LPS; HU, highly abundant nucleoid-associated histone-like protein; LPS, lipopolysaccharide; 4WJ, four-way junction.

In the section immediately above, data was presented to support the folding and dimerization of HU-B, LoodHU-B, LysrHU-B, and LoodLysrHU-B. Similarly, these DNA-binding site–ablated molecular species were also created using HU-A. As judged by the ability of these variants to elicit an electrophoretic mobility shift in 4WJ DNA, Figure 6 shows that HU-B, or HU-A, possessing either the canonical or noncanonical DNA binding site, is able to bind to 4WJ DNA. In contrast, HU-B or HU-A, lacking both sites is unable to bind to DNA. In Figure 6, control DNA (4WJ) is shown in lanes 1, 7, and 11. HU-B, LoodHU-B, and HU-B containing individual K83A and K86A mutations are all seen to bind 4WJ DNA (lanes 2–5) as is LysrHU-B (lane 6). However, LoodHU-B additionally lacking either of the K83 or K86 lysine residues, or both lysine residues (*i.e.*, partial and total LoodLysrHU-B variants), fails to bind to 4WJ DNA (lanes 8–10). Similarly, with HU-A and its LoodHU-A and LysrHU-A variants, it is seen that HU-A lacking K83, K86, or both lysine residues (*i.e.*, partial or total LysrHU-A) is still able to bind to 4WJ DNA, as would be expected, because of the retention of the canonical DNA-binding site. Interestingly, exactly as seen with HU-B, when the canonical DNA-binding site is absent (*i.e.*, LoodHU-A) and some or all of the noncanonical site is also absent, that is, when the variant additionally lacks either K83 or K86 or both lysine residues (*i.e.*, partial or total LoodLysrHU-A), no binding of 4WJ DNA is observed. From all of these data, it is clear that the presence of at least one of HU's two DNA-binding sites (the canonical loop or the noncanonical double-lysine cluster) is required by HU for binding of DNA. Compromise of either site is tolerated and the HU molecule still binds to DNA, but compromise of both sites through loop deletion, or removal of one or both lysine residues, is not tolerated, and there is no DNA binding by such variants. In the section below, we present evidence based on glutaraldehyde cross-linking experiments that ablation of the same two sites also completely abrogates LPS binding, whereas individual ablation of the sites does not elicit the same effect. In other words, we demonstrate below that the DNA-binding and LPS-binding abilities exist in both the canonical and noncanonical sites.

#### f-LPS binds to HU-B, LoodHU-B, and LysrHU-B but not to LoodLysrHU-B

We next explored the binding of f-LPS by HU-B and its three variants, LoodHU-B, LysrHU-B, and LoodLysrHU-B, using the glutaraldehyde crosslinking experiment (akin to the experiment shown earlier in Fig. 2*H*). This was performed to establish whether glutaraldehyde crosslinks LPS, on the one hand, and HU-B and its above variants, on the other hand, into large covalently crosslinked assemblies that fail to enter the resolving SDS-PAGE gel, remaining at the interface of the stacking and resolving gels. In Figure 6, *D*–*G*, lanes 5, 10, 14, and 17 show the electrophoretic migration of protein molecular weight markers of known size (mentioned at the right edge of the figure). Lanes 1, 6, 11 and 18 show the electrophoretic migration of HU-B, LoodHU-B, LysrHU-B, and LoodLysrHU-B, below the 14.4-kDa protein molecular weight marker. Lanes 2, 7, 12, and 19 show the crosslinking by glutaraldehyde of HU-B and all three variants of HU-B into dimers (just above the 18.4-kDa protein molecular weight marker) or tetramers (just above the 35-kDa protein molecular weight marker) and, in some lanes, also hexamers (around the 66.6-kDa bands). Lane 3 shows that glutaraldehyde crosslinks HU-B and f-LPS into aggregates that cannot enter the resolving gel, and lane 4 shows that when no glutaraldehyde is present, there is no crosslinking. Entirely similar results with LoodHU-B are seen in lanes 8 and 9, and with LysrHU-B in lanes 13 and 15. However, with LoodLysrHu-B, the lanes with and without glutaradehyde are nearly identical, displaying crosslinking of LoodLysrHU-B into dimers, and tetramers, due to HU–HU interactions, but not into the aggregates that are unable to enter the resolving gel, at the resolving–stacking gel interface because of the lack of HU–LPS interactions.

## Discussion

The *E. coli* NAPs, HU-A and HU-B, share ∼69% amino acid sequence identity. We have shown that both HU isoforms are capable of binding to free lipopolysaccharide (f-LPS), as well as to c-LPS on the outer membranes of bacteria. The HU possesses two types of DNA-binding sites. The first of these sites (the canonical site) was discovered concomitantly with the determination of the structure of the HU in complex with DNA. The second site (the noncanonical site) was discovered subsequently, based on small-angle X-ray scattering studies.

Using structural bioinformatics-based distance measurements, we showed that certain pairs of positively charged amino acid residues at each of these sites (*i.e.*, residues R58 and R61 at the canonical DNA-binding site and residues K83 and K86 at the noncanonical DNA-binding site) happen to be perfectly positioned for binding of the phosphate moieties present in the lipid-A head groups of LPS.

Using microscale thermophoresis (MST), DAS, dynamic light scattering, BLI, glutaraldehyde crosslinking, polarized microscope-based birefringence studies involving LCs, fluorescence spectroscopy, and quenching studies involving Trp-incorporating variants of HU, we showed that HU and LPS interact. In particular, using MST, we measured an affinity in the range of a few tens of micromolar (∼34 μM); however, we emphasize that this is only indicative and do not ourselves lay much store by this particular affinity measurement, mainly due to lack of confidence in the homogeneity of size of the commercially sourced LPS, and lack of knowledge about the fraction of the LPS present in micellar form (or the size of micelles) because most of our experiments, barring the light scattering experiments, were conducted using LPS concentrations in the post-CMC range of LPS concentrations.

Using fluorescence microscopy and fluorescence-based cytometry experiments, together with protein constructs in which fluorescent proteins were placed in fusion with HU, we established that f-HU binds to the surfaces of *E. coli* cells. We also showed that HU appears on the surfaces of cells overexpressing HU.

Using a combination of fluorescence-based cytometry experiments, in which we incubated f-HU with bacterial cells either with or without f-LPS or DNA, we further established that f-HU can cause the clumping of bacterial cells, and that f-LPS, DNA, and salt abrogate the clumping by progressively saturating HU's LPS-/DNA-binding sites.

Using genetic ablation of the canonical and noncanonical DNA-binding sites of HU, both individually and in combination, we showed that each site is capable of binding to both DNA, and LPS, but that HU is unable to bind to DNA or LPS when both types of sites are ablated. This establishes, in our view, that there is a complete physical coincidence of the DNA-binding and LPS-binding sites of the HU. In hindsight, this is not altogether surprising, considering that the lipid-A head group of LPS contains an arrangement of sugars in conjunction with phosphate groups (4-phospho-β-GlcN-(1,6)-α-GlcN-1-phosphate), which is somewhat akin to the sugars and phosphates in the backbone of DNA. In DNA, the phosphate groups are constituent parts of phosphodiester bonds, whereas in LPS, they are terminal phosphates. Thus, each phosphate group in DNA carries a single negative charge, whereas each of the two phosphate groups in LPS can carry either one, or two, negative charges, depending on the pH of the environment (which can rise to 9.0 in LB media). Furthermore, each sugar in DNA is a pentose (ribose) sugar, whereas each sugar in the lipid A head group of LPS is a hexose (glucosamine) sugar. So, the head group of lipid A in LPS is only notionally, or nominally, like DNA, and not really like DNA. The likeness is limited to LPS being capable of presenting phosphate groups to the DNA-binding site of a DNA-binding protein, in conjunction with sugar-like arrangements of carbon, hydrogen, and oxygen atoms. The important thing to note is merely that LPS can bind to the DNA-binding site of a DNA-binding protein by presenting sugar–phosphate moieties to the site, despite the differences in the natures of the sugars and the phosphates.

Our studies with *E. coli* cells thus indicate that HU is capable of acting as a glue that allows negatively charged cell surfaces (which could otherwise be expected to repel each other) to bind to positively charged multimers of the HU. We have shown that *E. coli* cells expressing HU tend to attach to each other after division, suggesting the involvement of some proteins on the cell surface (which could be HU). We have also shown that fusions of YFP/Venus and HU appear outside the cell, in association with the cell surface. In fact, it was this observation which first caused us to wonder about the presence of HU outside the cell and also about its possible association with the cell surface and its role in biofilms.

There is evidence that HU can appear outside the cell in the extracellular material that constitutes biofilms (5). There is also evidence that HU can be secreted by type IV pathways (11), or simply get disgorged by cells through explosive cell lysis, along with DNA (9). We propose that HU present in the extracellular medium in a developing biofilm binds to the surfaces of adjoining cells that are also in motion and that this results in uneven stresses upon bound cells that then become the cause for explosive cell lysis. In fact, videos of such lysis indicate that the expelled DNA (which turns into the e-DNA matrix of a biofilm) is bound by cells and dragged around by cells (9). We feel that this is because the expelled DNA is decorated with molecules of HU and that not all of the DNA-binding sites on such HU molecules are engaged in the binding of DNA, with some remaining vacant and available for interactions with the surfaces of bacterial cells. It is possible that such vacant DNA-binding sites bind to c-LPS on cell surfaces, allowing cells to bind to e-DNA through interactions with the DNA-bound HU. In fact, this has the potential for becoming a self-perpetuating process, such that each event of explosive cell lysis holds the potential of becoming the cause of the next event of explosive cell lysis, merely by making more e-DNA available for cells to bind to (and feel physically stressed by), as cells grow, divide, and move around, inside a bacterial colony. Indeed, we have shown evidence that exogenous addition of HU to bacterial cells in nonshaken cultures leads to the generation of large PI-binding, DNA-rich entities (*i.e.*, simulacrums of biofilms) in which bacterial cells are found to be embedded. This suggests that addition of exogenous HU can even begin the process of biofilm formation, with explosive cell lysis by growing and dividing bacteria possessing sites of weakness in their outer cell membranes and cell walls. There is evidence in the literature to suggest that addition of the HU reinforces the formation of biofilms (5), similar to our observations of addition of the HU causing embedment of cells in large matrices of DNA.

Thus, we emphasize that HU could be a central player in biofilms, (a) by being present in great abundance, (b) by being present everywhere upon the e-DNA matrix within biofilms, and (c) also by being able to bring together e-DNA and negatively charged surfaces of bacterial cells, as well as clump cells, as shown in this article. Therefore, we emphasize that the LPS-binding and cell-clumping abilities of HU, far from being interesting curiosities, could actually be central factors in the mechanism of association of *E. coli* cells to form biofilms, especially under conditions of exhaustion of nutrients and/or starvation because under such conditions, the death of a few cells (and the HU-bound DNA expelled therefrom) could, through feedback, rapidly scale up cell–cell associations, stresses upon cells, instances of DNA expulsion, participation of cells in clumping, and participation of cells in binding to the DNA-bound HU. LPS-binding by HU could thus cause cells to stick to each other, as we have shown, as well as cause cells to stick to bits of e-DNA (themselves networked through binding to multimeric forms of HU), to form the very foundations of biofilms.

Of course, it is reasonable to assume that there would be likely constant competition between e-DNA, and LPS, for binding to HU's DNA-binding sites. We have shown that (i) DNA-bound HU binds less to c-LPS (to cause less clumping), (ii) f-LPS-bound HU also binds less to c-LPS (to cause less clumping), and (iii) salt interferes electrostatically with the interactions of HU with c-LPS (to cause less clumping). It is already known that antibodies raised against the DNA-binding tips (i.e, the canonical beta hairpins) of HU cause dislodgement of bacteria from biofilms (6). All of the available evidence, therefore, points toward involvement of HU in the formation and stabilization of biofilms. It may be noted that all bacteria have negatively charged surfaces and that if they do not possess c-LPS on their surfaces, they possess another negatively charged sugar–phosphate arrangement involving a different molecule, lipoteichoic acid, which also has sugar-phosphate moieties. Therefore, because all bacteria possess HU, this protein could be a glue for biofilms formed by all gram-negative and gram-positive bacteria.

## Experimental procedures

### Bacterial strains, media, plasmids, and protein expression

The XL-1 Blue strain of *E. coli* K-12 was used for all experiments. Cells were grown using the LB medium. Expression of all HU-based proteins (including engineered variants of the HU) was carried out using these cells, for which cells were transformed by pQE-30 (Qiagen) expression vectors incorporating genes encoding proteins of interest inserted in the vector's multiple cloning sites. Proteins were purified using standard Ni-NTA affinity-purification IMAC methods (Qiagen QIAexpressionist) under nondenaturing conditions. Attached genomic nucleic acid fragments and bound proteins were removed from the HU during purification through washing of all Ni-NTA-bound HU protein forms (WT and mutants/variants) with 15 column volumes of 2 M NaCl, leading to dissociation and removal of all contaminant DNA and associated proteins.

### Recombinant (engineered) proteins and other reagents

Genes encoding HU-A and HU-B were PCR-amplified from *E. coli* K-12 genomic DNA and cloned into the multiple cloning sites of the pQE-30 vector between the Bam HI and Hind III restriction enzyme sites, such that the two proteins (and all of their mutants, deletion or truncation variants, and fluorescent protein fusions, as described below) could be produced with an N-terminal 6xHis affinity tag facilitating purification: (i) HU-A WT; (ii) HU-B WT; (iii) RFP–HU-A, that is, HU-A with RFP (tag-RFP) present in fusion at HU's N-terminus, without any linker; (iv) Venus–HU-B, that is, HU-B with yellow fluorescence protein/Venus present in fusion at HU's N-terminus, without any linker; (v) Trp-containing HU-B mutant, F47W HU-B; (vi) Trp-containing HU-B mutant, F79W HU-B; (vii) LoodHU-B, that is, HU-B in which residues 52 to 74 (beta hairpin loop containing HU's canonical DNA-binding site residues) are ablated/deleted and replaced by an 11-amino acid-long glycine–serine linker (N-SGGGGSGGGGS-C); (viii) LysrHU-B, that is, HU-B in which residue mutations K83A and K86A (at HU's noncanonical DNA-binding double-lysine cluster) effect lysine residue by alanine residues; and (ix) LoodLysrHU-B, that is, HU-B combining the LoodHU-B and LysrHU-B mutations.

All mutants and variants were verified through DNA sequencing. Oligonucleotides used for PCR and splicing by overlap extension PCR-based mutagenesis or protein fusions were sourced from Integrated DNA Technologies or Sigma. LPS was sourced from Sigma (Catalog No. L-2630-25MG). 4WJ DNA was sourced in the form of four independent oligonucleotides from Integrated DNA Technologies or Sigma through contract synthesis and assembled into 4WJ DNA through addition of the following four oligonucleotide strands to each other in equimolar amounts (strand 1: 5′-CCCTATAACCCCTGCATTGAATTCCTGTCTGATAA-3′; strand 2: 5′-GTAGTCGTGATAGGTGCAGGGGTTATAGGG-3′; strand 3: 5′-AACAGTAGCTCTTAATTCGAGCTCGCGCCCTATCACGACTA-3′; strand 4: 5′-TTTATCAGACTGGAATTCAAGCGCGAGCTCGAATAAGAGCTACTGT-3′). Restriction enzymes used for recombinant DNA work were sourced from Thermo Fisher Scientific. DNA polymerase and ligase enzymes were sourced from New England Biolabs. All other media, chemicals and reagents, including poly-D-lysine (Catalog No. P6407-5MG) were sourced from Hi-Media or Sigma, or from individual manufacturers of instruments for consumables associated with specific instruments.

### Instrument-based spectroscopic, microscopic, cytometric, and other analyses

UV-visible absorption spectral measurements for protein concentration estimation were collected on a Varian 50-Bio spectrophotometer, using a microcuvette with a path length of 0.3 cm for standard measurements. For DAS measurements, a tandem quartz cuvette of 1-cm path length was used, incorporating two tandem compartments of 0.45-cm path length each. The two tandem compartments were filled with 1 ml of 2 mg/ml LPS, and 1 ml of 10-μM RFP–HU-A, respectively. Mixing of the contents was performed through inversion of the cuvette after collection of the baseline, as described earlier (24, 25). Fluorescence spectral measurements for collection of fluorescence emission spectra and fluorescence quenching upon LPS binding to Trp-containing variants of HU-B were made using 295-nm excitation and 5-nm excitation and emission slid-widths on a Varian Eclipse spectrofluorometer, using a 0.3 × 0.3 cm quartz cuvette, and HU-B (F47W or F79W) protein of 0.65 mg/ml concentration and LPS concentrations varying from 0.14 to 0.56 mg/ml. CD spectra were collected to estimate the protein secondary structural content using a BioLogic MOS-500 spectrometer, using a cuvette of 0.1-cm path length, and protein concentrations in the range of 0.25 to 0.35 mg/ml. Dynamic light scattering measurements of protein size were made using a Wyatt Dawn 8+ instrument and ASTRA software, using a protein concentration of 0.5 mg/ml and an LPS concentration of 2 mg/ml. BLI measurements were made using a ForteBio BLItz instrument and Ni-NTA-derivatized tips from ForteBio to examine interactions between the tip-bound HU and LPS, using an HU-B concentration of 6 μM and LPS of 1 mg/ml concentration, and PBS of pH 7.4. MST measurements were made on a Nanotemper Monolith NT-115 instrument with 16 capillaries, with fluorescence excitation through a 550-nm laser to examine protein diffusion as a function of LPS to estimate HU–LPS binding. The RFP–HU-A protein concentration used for 250 nM, and the LPS concentration was 2.5 mg/ml in the first capillary and serially diluted through halving of concentrations over the remaining 15 capillaries. The temperature jump involved heating by 2 °C. Flow cytometry measurements of bacterial clump sizes and the fluorescence associated with binding of RFP–HU-A to bacteria were made on a Becton-Dickinson Accuri C-6 instrument. Gel filtration chromatography measurements of the protein elution volume and molecular weight for HU-B and its variants were made using Superdex-75 10/300GL columns on an AKTA Purifier-10 GE Healthcare instrument. Fluorescence microscopy images were collected to examine PI-labeled permeabilized *E. coli* cells embedded in a DNA matrix using a Nikon Eclipse Ti-u microscope. Confocal fluorescence microscopy images were acquired using a table-top Olympus FluoView FV10i microscope to examine RFP–HU association with *E. coli* of the XL-1 Blue strain. Wide-field fluorescence and DIC deconvolution microscopy images and videos were collected using a wide-field, high-resolution, DeltaVision Deconvolution microscope [Model DV Elite, GE Healthcare] equipped with solid-state illumination and a 1.4-megapixel monochrome CCD camera [CoolSnap HQ2, Photometrics]. Analytical electrophoresis was performed using a Bio-Rad Mini-Protean Tetra vertical electrophoresis set up for glutaraldehyde-based crosslinking studies of LPS with HU-B (and variants) using 15% SDS-PAGE gels and Coomassie Blue G-250 protein staining according to standard methods, and using a Bio-Rad Wide Mini-Sub Cell GT submarine set up for electrophoretic mobility shift assays involving 4WJ DNA and HU-B (and variants) using 0.7% or 1% agarose gels and ethidium bromide DNA staining. Protein structural and distance analyses/representations used PYMOL software from Schrodinger. LC birefringence experiments were conducted as earlier described (26). The concentration of protein used was 0.5 mg/ml. All other details are described in the reference provided, and the preparation of the LCs was performed exactly as described in the reference, which also immobilized LPS (bacterial endotoxin) on the LCs for diagnostic detection of protein binding to endotoxin. Details of the control experiments eliciting bright and dark fields and of the assay monitoring disruption of organization of LPS upon the LC by binding of the HU have been described in Results, sufficiently to understand the results and interpretation.

## Data availability

All data relating to this article are contained in the article and the file presenting supporting information.

## Supporting information

This article contains supporting information.

## Conflict of interest

The authors declare that they have no conflicts of interest with the contents of this article.
