# Supplementary material for: The DNA-binding protein HU is a molecular glue that attaches bacteria to extracellular DNA in biofilms
Source: J Biol Chem. 2021 Mar 11;296:100532. doi: 10.1016/j.jbc.2021.100532 (PMC8063757; doi:10.1016/j.jbc.2021.100532)
Supplement: Figures S1 to S8 [file mmc1.docx]

**SUPPORTING INFORMATION FOR**

**The DNA-binding protein HU is a molecular glue that attaches bacteria to extracellular DNA in biofilms**

Bhishem Thakur, Kanika Arora, Archit Gupta and Purnananda Guptasarma^*^

Centre for Protein Science, Design and Engineering (CPSDE),

Department of Biological Sciences,

Indian Institute of Science Education and Research (IISER) Mohali,

Knowledge City, Sector-81, SAS Nagar, Punjab 140306

* Author to whom correspondence may be addressed ([guptasarma@iisemohali.ac.in](mailto:guptasarma@iisemohali.ac.in)); Cell: +91-9815417265; Tel : +91-172-2293151; Fax : +91-172-2240266, +91-172-2240124; website : [www.guptasarmalab.in](http://www.guptasarmalab.in)

**
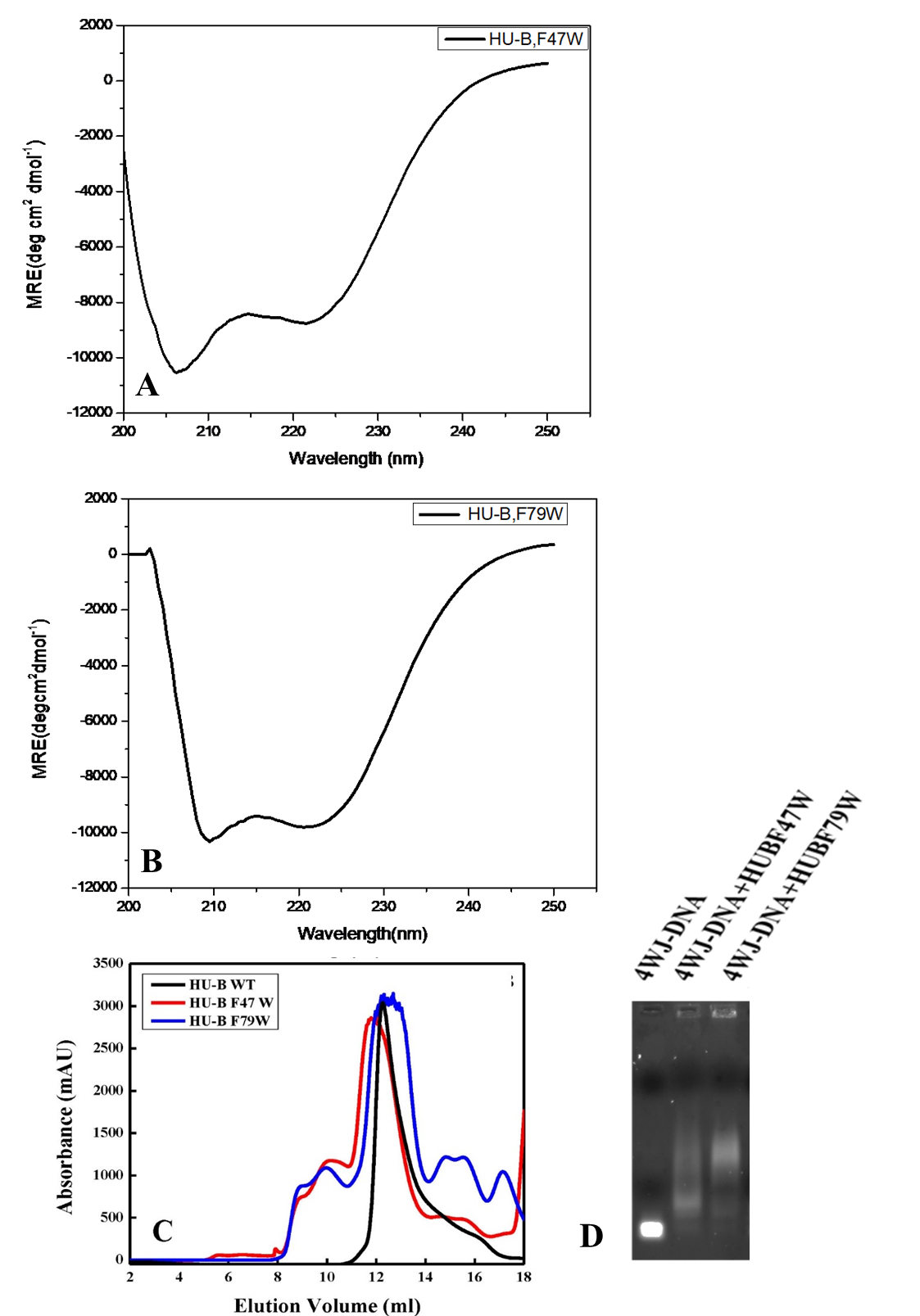
**

**Supplementary Fig. 1 | CD and gel filtration chromatographic behaviour of tryptophan-substituted mutants of HU-B**. **A,** CD spectrum of F47W HU-B, **B,** CD spectrum of F79W HU-B, **C,** Gel filtration chromatograms of F47W HU-B (red), F79W HU-B (blue) and wild-type HU-B (black), **D,** Electrophoretic mobility shift assay gel electrophoresis showing 4WJ DNA, 4WJ DNA with F47W HU-B, and 4WJ DNA with F79W HU-B.


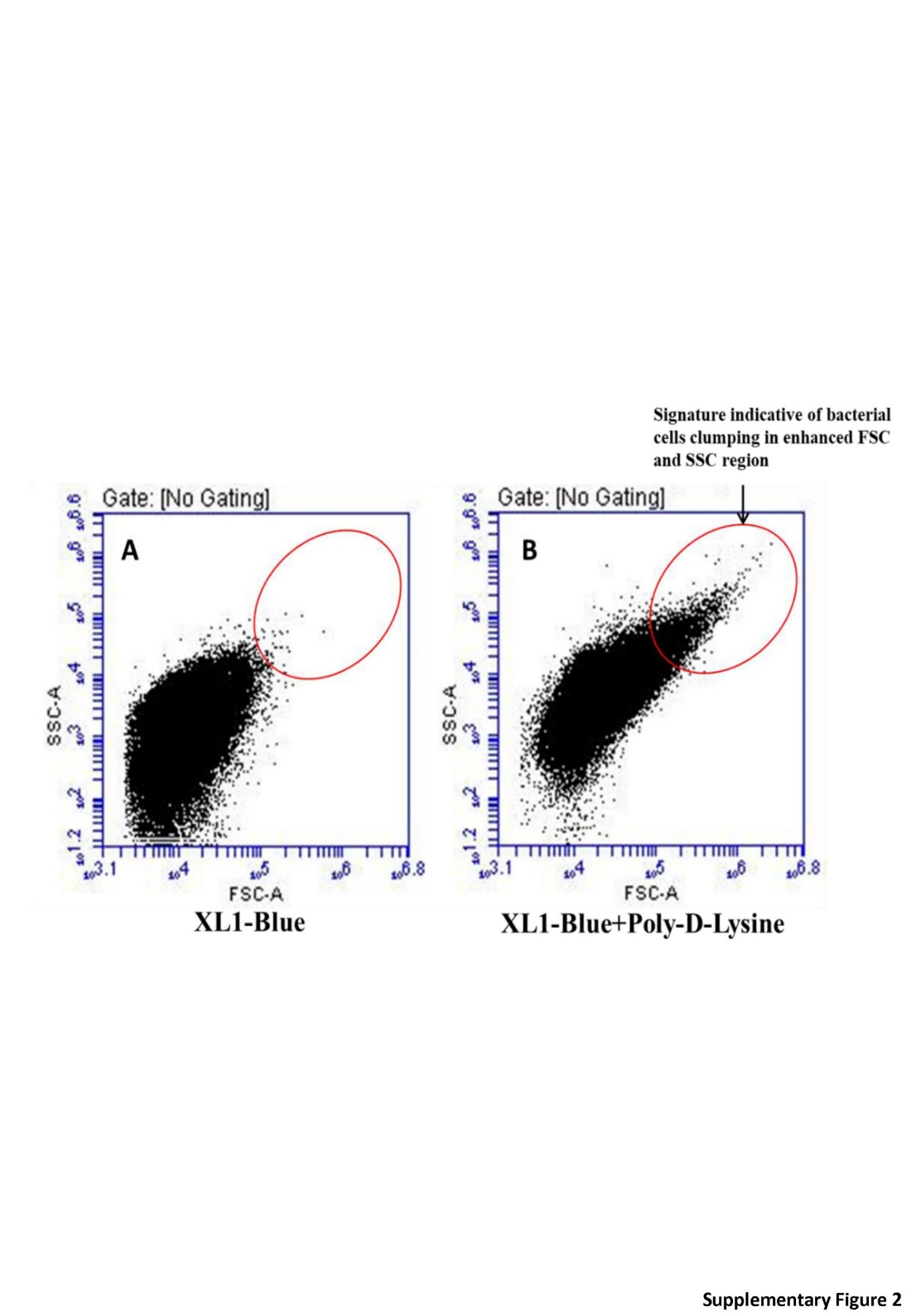


**Supplementary Fig. 2 | Poly-D-lysine mediated bacterial cells clumping**. **A,** Scatter plot of for the untreated XL1-Blue cells plotted between FCS-A, SSC-A, X and Y axis respectively. **B,** Scatted plot of for the XL1-Blue cells after treating with poly-D-lysine.

**A**

**B**

**Supplementary Fig. 3 | Presence of Venus-HU-B on the surface of expressing *E. coli* MG1655 cells**. **A,** Click on the above Windows Media Player video tab to play a video of an elongated (non-separated) syncytial *E.coli*(strain MG1655) cell expressing Venus-HU, in which the progressive viewing of different z-axial depths reveals that there is a halo of Venus-HU-B around the surface of the cell which is distinct from the Venus-HU-B present inside the cell in association with the cell’s chromosomal nucloied. The cell is on agar. The sections of the halo around the cell which are above the agar are much more distinctly visible than the sections in association with the agar. **B,** syncytial cell expressing Venus-HU-B breaking to generate smaller filament and individual cells displaying a tendency to stick to each other along their surfaces.

*
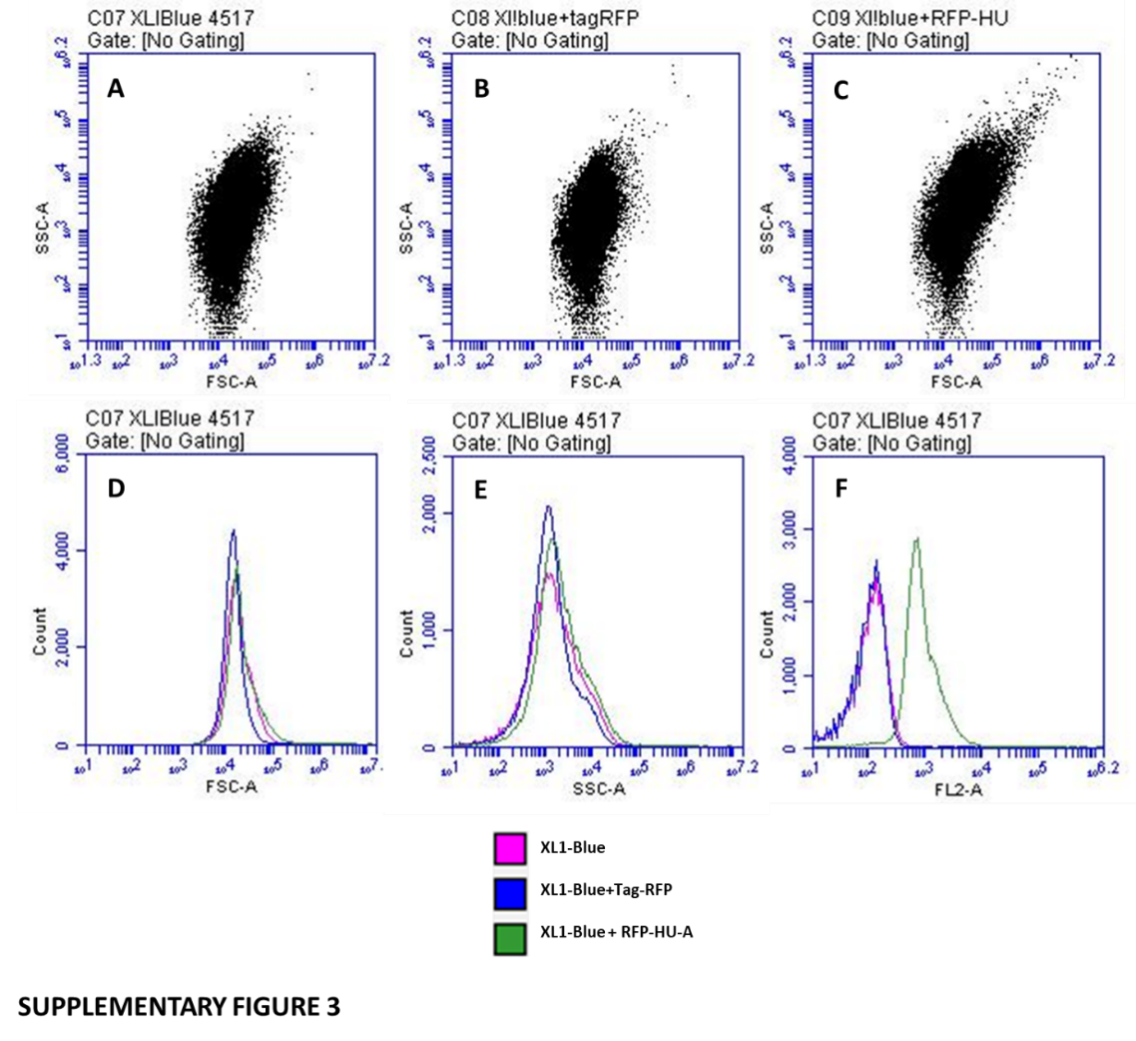
*

**Supplementary Fig. 4 | Tag-RFP-HU-A binds to the c-LPS without causing much clumping proved by flow cytometry**. **A, B, C,** Scatter plot of for the untreated XL1-Blue cells and the cells incubated with Tag-RFP and Tag-RFP-HU-A proteins. **D**, **E**, Overlay of the histogram for three sets of bacterial cell counts plotted with FSC-A and SSC-A values. **F,** Overlay of the histograms of three sets of the cells to compare the amount of the fluorescence associated with them.


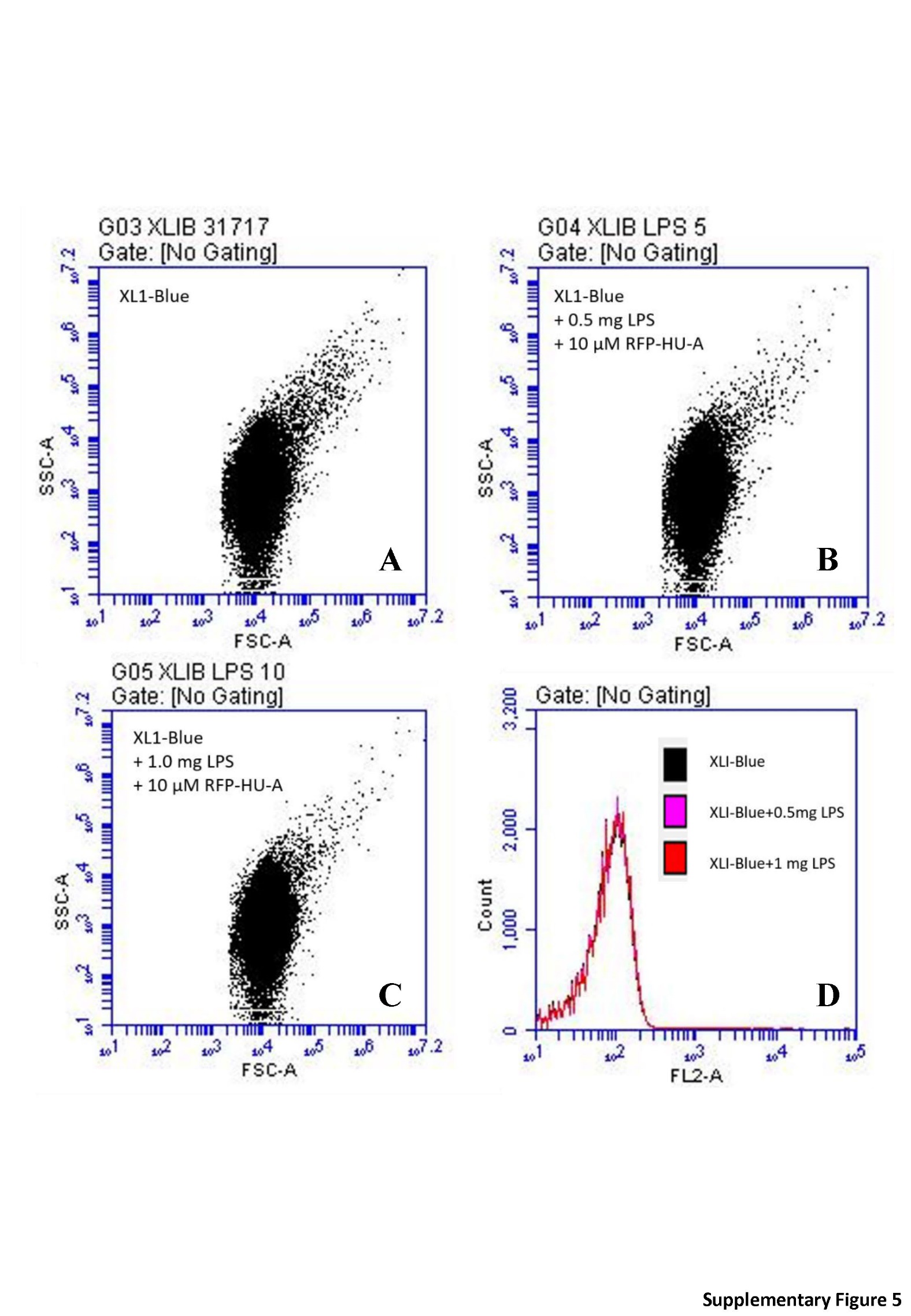


**Supplementary Fig. 5| Inhibition of c-LPS-HU-c-LPS interactions (*E. coli* clumping) through pre-incubation of RFP-HU-A with f-LPS.** f-LPS dose-dependent reduction in intensity of streaks in scatter plots derived from flow cytometry of *E. coli* cells, with monitoring of forward scatter *versus* side scatter using **A,** control XL1-Blue cells, **B,** XL1-Blue cells treated with 10 µM RFP-HU-A pre-treated with 0.5 mg/ml f-LPS, **C,** XL1-Blue cells treated with 10 µM RFP-HU-A pre-treated 1.0 mg/ml f-LPS. **D,** combined overlay for all cells with (and without) pre-treatment of RFP-HU-A with f-LPS; saturation of binding sites on HU-A by f-LPS lead to lack of RFP-HU-A binding to cells.


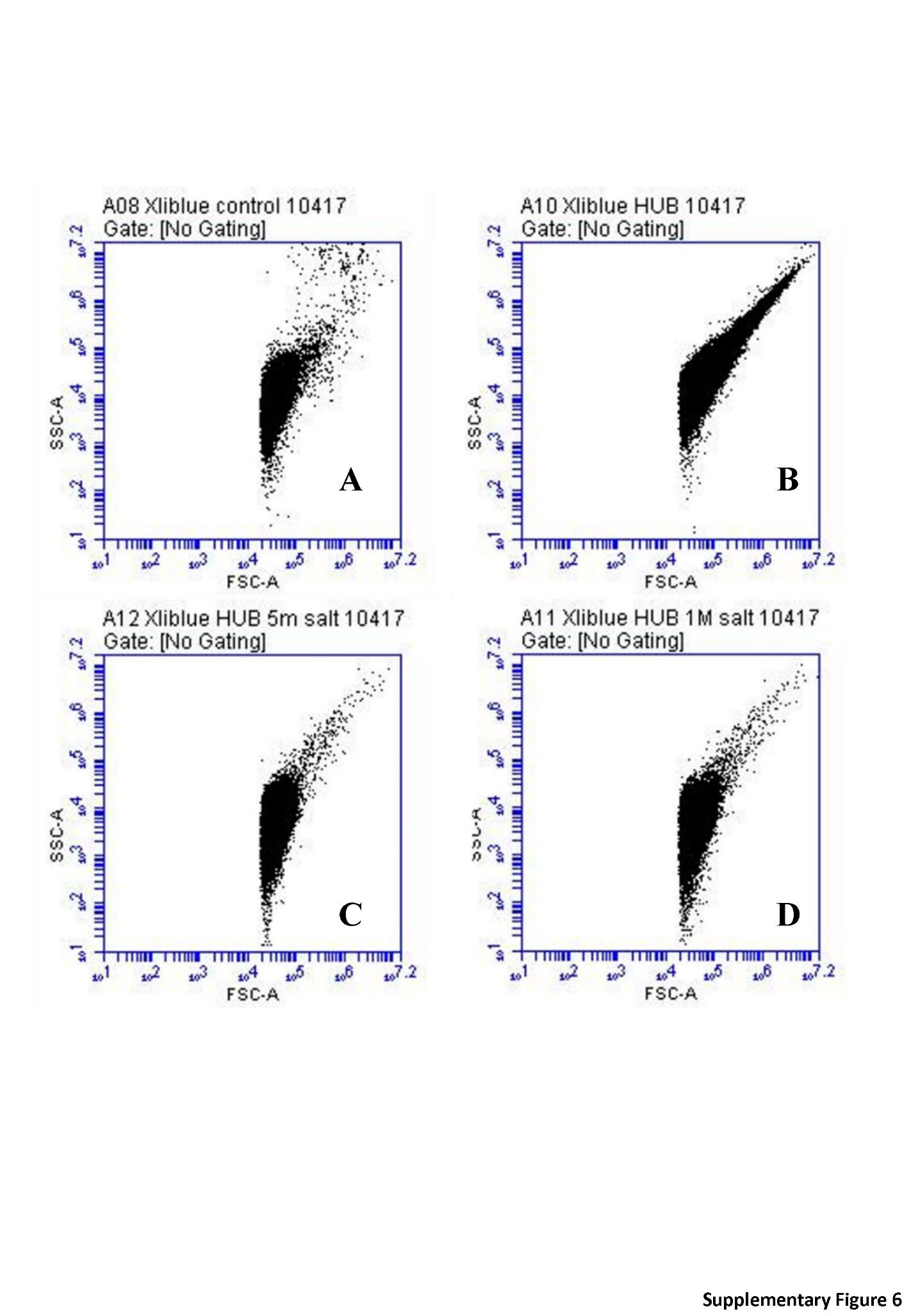


**Supplementary Fig. 6| Inhibition of c-LPS-HU-c-LPS interactions (*E. coli* clumping) through pre-incubation of HU-B with NaCl.** NaCl dose-dependent reduction in intensity of streaks in scatter plotsderived from flow cytometry of *E. coli* cells, with monitoring of forward scatter *versus* side scatter using **A,** control XL1-Blue cells, **B,** XL1-Blue cells treated with HU-B, **C,** XL1-Blue cells treated with HU-B in presence of 0.5 M NaCl. **D,** XL1-Blue cells treated with HU-B in presence of 1.0 M NaCl.


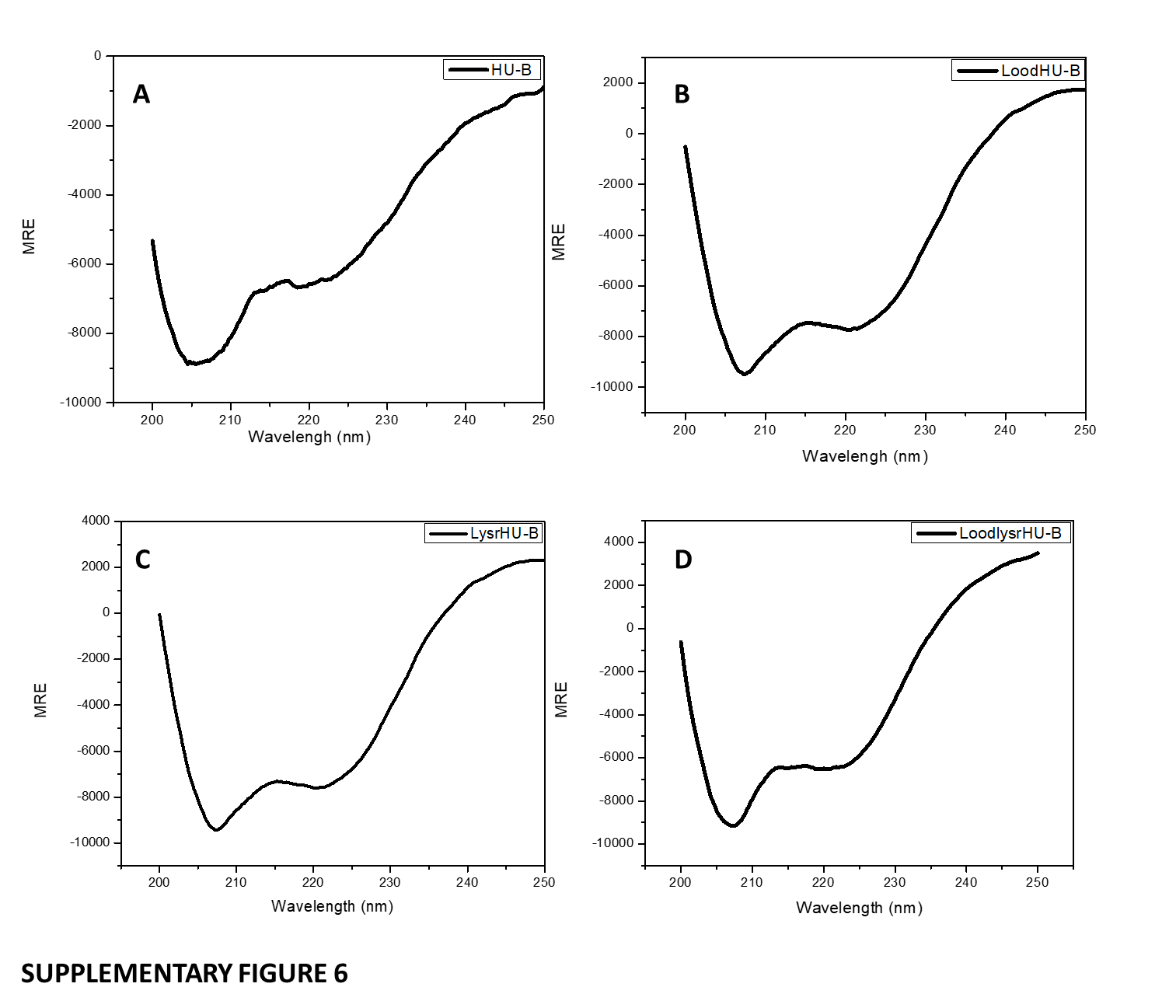


**Supplementary Fig. 7| Circular Dichroism (CD) spectra of HU and its DNA-binding site-ablated variants. A,** HU-B. **B,** LoodHU-B. **C,** LysrHU-B. **D,** LoodLysrHU-B.

*
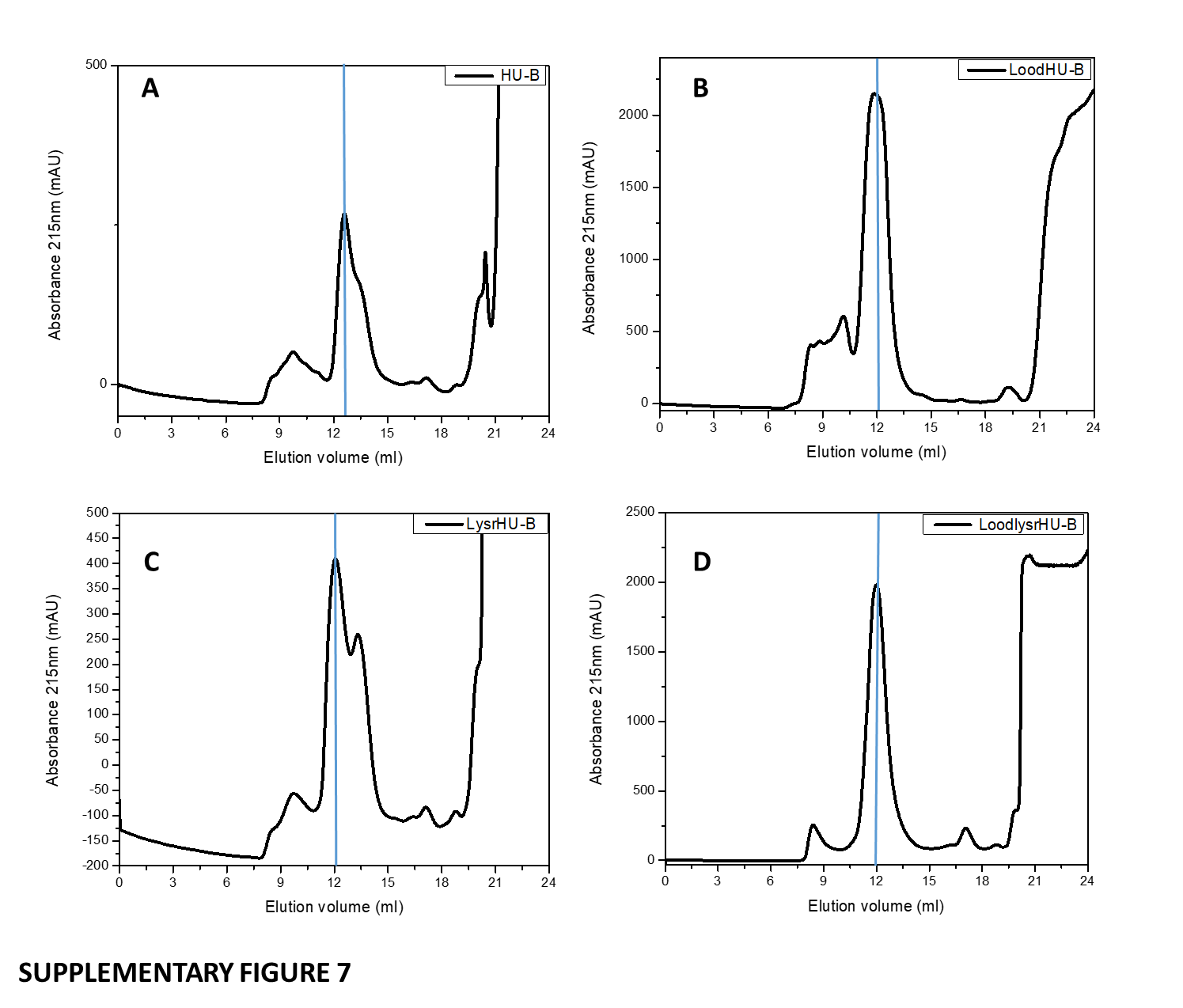
*

**Supplementary Fig. 8| Gel filtration chromatograms of HU and its DNA-binding site-ablated variants. A,** HU-B. **B,** LoodHU-B. **C,** LysrHU-B. **D,** LoodLysrHU-B.
